# Plasticity in gene transcription explains the differential performance of two invasive fish species

**DOI:** 10.1111/eva.12463

**Published:** 2017-04-25

**Authors:** Kyle W. Wellband, Daniel D. Heath

**Affiliations:** ^1^ Great Lakes Institute for Environmental ResearchUniversity of WindsorWindsorONCanada; ^2^ Department of Biological SciencesUniversity of WindsorWindsorONCanada

**Keywords:** biological invasions, gene expression, nonindigenous species, phenotypic plasticity, round goby, tubenose goby

## Abstract

Phenotypic plasticity buffers organisms from environmental change and is hypothesized to aid the initial establishment of nonindigenous species in novel environments and postestablishment range expansion. The genetic mechanisms that underpin phenotypically plastic traits are generally poorly characterized; however, there is strong evidence that modulation of gene transcription is an important component of these responses. Here, we use RNA sequencing to examine the transcriptional basis of temperature tolerance for round and tubenose goby, two nonindigenous fish species that differ dramatically in the extent of their Great Lakes invasions despite similar invasion dates. We used generalized linear models of read count data to compare gene transcription responses of organisms exposed to increased and decreased water temperature from those at ambient conditions. We identify greater response in the magnitude of transcriptional changes for the more successful round goby compared with the less successful tubenose goby. Round goby transcriptional responses reflect alteration of biological function consistent with adaptive responses to maintain or regain homeostatic function in other species. In contrast, tubenose goby transcription patterns indicate a response to stressful conditions, but the pattern of change in biological functions does not match those expected for a return to homeostatic status. Transcriptional plasticity plays an important role in the acute thermal tolerance for these species; however, the impaired response to stress we demonstrate in the tubenose goby may contribute to their limited invasion success relative to the round goby. Transcriptional profiling allows the simultaneous assessment of the magnitude of transcriptional response as well as the biological functions involved in the response to environmental stress and is thus a valuable approach for evaluating invasion potential.

## 1 Introduction

In recent decades, there has been renewed interest in phenotypic plasticity as a mechanism that facilitates species persistence in novel and changing environments (Ghalambor, McKay, Carroll, & Reznick, 2007). Phenotypic plasticity is defined as the ability of organisms with identical genotypes to alter a specific aspect of their phenotype, either transiently or permanently, in response to environmental factors (West‐Eberhard, 2003). Traditionally regarded as a source of unpredictable phenotypic variance (e.g., Wright, 1931), plasticity was believed to retard evolution by natural selection by obscuring adaptive genetic variation from selective pressures. However, the ability to alter phenotype in an environmentally dependent manner may be advantageous for organisms experiencing variable environments if the phenotypic changes provide a fitness advantage (Schlichting & Smith, 2002). Not surprisingly, both empirical and theoretical considerations of plasticity have demonstrated conditions where plasticity is adaptive (provides a fitness advantage; Price, Qvarnstrom, & Irwin, 2003), demonstrated plasticity's role in facilitating genetic adaptation through genetic accommodation (West‐Eberhard, 2003) and distinguished between plasticity that is adaptive (beneficial for an organism's fitness but not a product of selection) and plasticity that is an adaptation (beneficial for an organism's fitness and has been shaped by natural selection; Gotthard & Nylin, 1995). Plasticity that improves an organism's fitness is clearly an important trait for organisms experiencing environmental challenges such as those experienced when organisms colonize novel environments.

Biological invasions expose organisms to novel environments and provide an excellent opportunity to study the role of adaptive plasticity in population establishment, persistence, and expansion. Blackburn et al. (2011) developed a conceptual model to describe the invasion process as a series of barriers and stages that a species must pass through to be classified as invasive. Thus, a highly successful invasive species is not just one that survives and establishes in a non‐native region but one that expands its range throughout the non‐native region (Blackburn et al., 2011). Plasticity certainly plays a role in the survival of nonindigenous species during the “transport” and “establishment” stages of an introduction when environmental changes will be rapid and before evolutionary responses can occur; however, plasticity may also be critically important for the postestablishment range expansion that characterizes highly successful invasions. Species may rapidly evolve elevated plasticity to produce an optimal, yet responsive, phenotype during the range expansion phases of an invasion (Lande, 2015). This rapid increase in plasticity is then followed by assimilation of these traits by selection on standing genetic variation and relaxed selection for plasticity as populations stabilize (Lande, 2015). The role of plasticity in providing fitness advantages to organisms experiencing novel environments has generated interest in whether successful invaders are more plastic than unsuccessful invaders; however, support for the hypothesis that invaders are more plastic than noninvaders is inconsistent (Davidson, Jennions, & Nicotra, 2011; Godoy, Valladares, & Castro‐Díez, 2011; Palacio‐López & Gianoli, 2011). Phenotypic plasticity is expected to change through the stages of an invasion and the inconsistent support for plasticity as an important mechanism driving invasion success is likely a result of the varied amount of time since invasion for species included in these studies (Lande, 2015). As a result, direct tests of the hypothesis that more successful invaders have greater plasticity must compare species with similar invasion timing and histories.

There is a growing body of the literature implicating gene expression variation as a mechanism that facilitates plastic phenotypic responses to environmental change (Aubin‐Horth & Renn, 2009; Schlichting & Smith, 2002). Gene expression is a phenotype that responds to environmental cues and is the mechanistic basis for different phenotypes expressed by different types of cells, tissues, and organisms (Wray et al., 2003). Gene transcription, the initial step in gene expression, has shown the capacity to evolve both changes in constitutive expression (Whitehead & Crawford, 2006) and altered responses to environmental cues (Aykanat, Thrower, & Heath, 2011). As a key regulator of the physiological status of organisms, there has been an increased focus on the role of gene transcription as a mechanism underlying plastic traits in wild populations; examples include salinity tolerance (Lockwood & Somero, 2011; Whitehead, Roach, Zhang, & Galvez, 2012), immune function (Stutz, Schmerer, Coates, & Bolnick, 2015), long‐term thermal acclimation (Dayan, Crawford, & Oleksiak, 2015), and acute thermal tolerance (Fangue, Hofmeister, & Schulte, 2006; Quinn, McGowan, Cooper, Koop, & Davidson, 2011). Increased thermal tolerance has been linked to invasion success (Bates et al., 2013). Widespread transcriptional changes in response to both acute exposure and long‐term acclimation to thermal stress have been documented in a diverse array of taxa including plants, yeast, invertebrates, fish, and mammals (Logan & Somero, 2011; Smith & Kruglyak, 2008; Sonna, Fujita, Gaffin, & Lilly, 2002; Sørensen, Nielsen, Kruhøffer, Justesen, & Loeschcke, 2005; Swindell, Huebner, & Weber, 2007) indicating that transcriptional plasticity plays an important and evolutionary conserved role in both short‐ and long‐term responses to altered temperature (López‐Maury, Marguerat, & Bähler, 2008). Given the important role of transcriptional plasticity in mediating physiological changes associated with thermal stress, the question arises: Do successful invasive species exhibit higher transcriptional plasticity in response to thermal stress? Indeed there is some evidence that transcriptional plasticity may be a feature of successful biological invasions as an increased capacity for transcriptional response to temperature exposure has also been observed in a highly successful marine invader *Mytilus galloprovincialis* compared to its native conger *Mytilus trossulus* on the west coast of North America (Lockwood, Sanders, & Somero, 2010).

Understanding attributes that make invaders successful is a critical aspect of the management of invasive species (Kolar & Lodge, 2001). Ideally, experiments testing the importance of invasive traits should compare congeners exhibiting a successful and failed invasion in the same environment (Kolar & Lodge, 2001); however, this presents the logistical challenge of studying organisms that do not exist (failed invader). In this study, we take advantage of a nearly analogous instance of a highly successful invasion (as determined by extent of range expansion) and a less successful invasion between two phylogenetically and invasion history paired species in the Laurentian Great Lakes of North America to test the hypothesis that more successful invasive species are more transcriptionally plastic than less successful invasive species.

**Figure 1:**
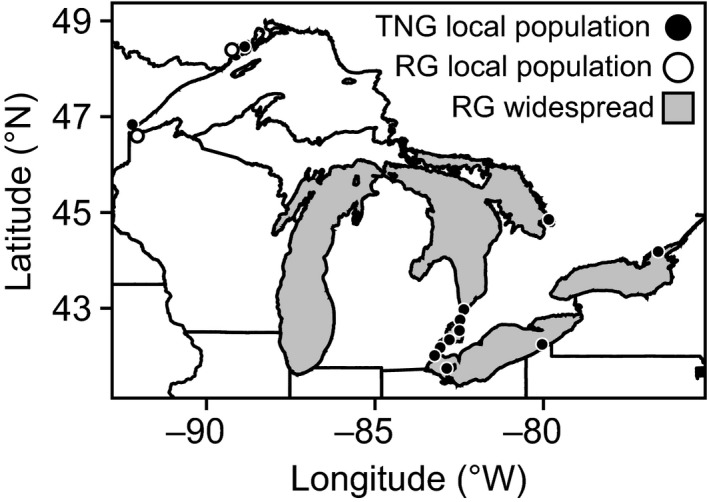
Map of the Laurentian Great Lakes contrasting the postinvasion dispersal and distribution of round and tubenose gobies. Round goby are widespread throughout lakes Michigan, Huron, Erie, and Ontario with local populations in Lake Superior (open circles). Local established populations of tubenose goby indicated by black circles. Distribution data from U.S. Geological Survey (2016)

Round goby (*Neogobius melanostomus*, Pallas) and tubenose goby (*Proterorhinus semilunaris*, Heckel) are two species of fish from the family Gobiidae that possess overlapping geographic ranges and habitat in their native Ponto‐Caspian region of Eastern Europe. These species were both first detected in North America in the St. Clair River in 1990 (Jude, Reider, & Smith, 1992), presumably introduced via ballast water carried by cargo ships originating from the Black Sea (Brown & Stepien, 2009). Since introduction, round goby have spread throughout the entire Great Lakes basin and reached high population densities in many areas, while tubenose goby have mostly remained geographically restricted to the Huron–Erie corridor near the site of initial introduction and occur at low population densities (Figure 1). There is limited information about factors that may have differentially restricted range expansion for these species. Round goby have small home ranges (~5 m^2^; Ray & Corkum, 2001) and typically do not disperse more than 500 m on their own (Lynch & Mensinger, 2012; Wolfe & Marsden, 1998). Similar information is unavailable for tubenose goby in the Great Lakes; however, it is difficult to imagine that the dispersal attributes described above would provide round goby with an advantage that would explain the differential range expansion and impact. The presence of both species in Lake Superior (Figure 1) suggests that differences in secondary transport due to shipping vectors within the Great Lakes are unlikely to explain the differential range expansion. Tubenose goby are slightly smaller on average than round goby (maximum total length in the Great Lakes: TNG ~ 130 mm, RG ~ 180 mm; Fuller, Benson, et al. 2017; Fuller, Nico, et al. 2017), but this does not appear to result in large differences in fecundity (MacInnis & Corkum, 2000b; Valová, Konečná, Janáč, & Jurajda, 2015).

Differences in phenotypic plasticity may explain the difference in invasion performance of round and tubenose goby. Round goby exhibit greater dietary plasticity compared to tubenose goby (Pettitt‐Wade, Wellband, Heath, & Fisk, 2015). Thermal performance curves suggest that round goby has a broad thermal tolerance (Lee & Johnson, 2005). While similar curves are unavailable for tubenose goby, they have similar standard and resting metabolic rates at near optimum temperatures (O'Neil, 2013; Xin, 2016) but reduced performance at temperature extremes. Tubenose goby have a decreased upper critical thermal limit (31.9°C) compared with round goby (33.4°C; Xin, 2016) and exhibit higher standard metabolic rates at elevated temperatures (O'Neil, 2013) that may indicate a narrower range of temperature tolerance than round goby. In addition to the difference in performance at elevated temperatures, the expansion and impact of invasive fish species in the Great Lakes are also typically limited by cold temperature tolerance (Kolar & Lodge, 2002); however, specific critical limits are unavailable for these species.

Changes in gene transcription underpin many adaptive responses to acute and long‐term temperature exposure (e.g., Logan & Somero, 2011). To investigate the genetic mechanisms that underlie apparent differences in thermal tolerance, we use RNA sequencing (RNAseq) to characterize the liver transcriptomes of round and tubenose goby in response to acute exposure to increased and decreased temperatures. Liver tissue is a key regulator of a fish's metabolic processes and is known to play an important role in molecular reprogramming of metabolism in response to acute stressors (Wiseman et al., 2007). We predict that (i) the round goby will show generally higher transcriptional plasticity (more genes responding and at higher magnitudes of transcriptional change) across the liver transcriptome and (ii) the observed transcriptional variation will have greater functional relevance for maintaining homeostatic function in the round goby relative to the tubenose goby. Transcriptional profiling has enormous potential for applications in conservation biology (e.g., He et al., 2015; Miller et al., 2011) and a characterization of the evolutionary processes driving variation in transcription in invasive species may extend that utility to invasion biology.

## 2 Methods

### 2.1 Sample collection and experimental design

Round and tubenose gobies were collected in the first week of October 2014 from the Detroit River using a 10‐m beach seine net. Although we did not directly age the fish, they ranged in size from 48 to 69 mm total length, indicating that most were age‐1 with possibly some age‐2 for the larger round goby, although they are typically absent in samples by October (MacInnis & Corkum, 2000a). No individuals were reproductively mature as determined by the absence of developed gonads during tissue dissection, all fish appeared healthy and no fish died during the experimental procedures. Gobies were immediately transferred to the aquatics facility at the Great Lakes Institute for Environmental Research in aerated coolers where they were immediately placed into one of three different water temperature tanks (five fish per tank). Each temperature treatment consisted of paired 10‐L tanks (one for round goby and one for tubenose goby) connected to a recirculation system that aerated the water and controlled water temperature. The three temperature conditions were the following: (i) control: ambient water conditions in the aquatics facility (18°C) that was drawn from the Detroit River immediately upstream from the sampling site (<100 m) and reflects the temperature both species were exposed to prior to sampling, (ii) high‐temperature challenge: increasing the water temperature 2°C per hour from ambient to 25°C, and (iii) low‐temperature challenge: decreasing the water temperature 3°C per hour from ambient to 5°C. Temperatures were chosen to represent a range of temperatures potentially experienced during range expansion from the St. Clair River throughout the extent of the North American range expansion of round goby but less extreme than known critical thermal limits for these species (round goby: 33.4°C and tubenose goby 31.9°C, Xin, 2016). Once the treatment temperature was reached, fish were held in these conditions for 24 hr after which they were humanely euthanized in an overdose solution of tricaine methylsulfonate (200 mg/L MS‐222, Finquel, Argent Laboratories, Redmond, WA). All fish (five per treatment, per species) were weighed and measured and liver tissue was immediately dissected, preserved in a high salt solution (700 g/L ammonium sulfate, 25 mM sodium citrate, 20 mM ethylenediaminetetraacetic acid, pH 5.2), and stored at −20°C.

### 2.2 RNA sequencing and de novo transcriptome assembly

RNA was extracted from liver tissue using TRIzol^®^ reagent (Life Technologies, Mississauga, ON) following the manufacturer's protocol. RNA was dissolved in sterile water and treated with TURBO^™^ DNase (Life Technologies, Mississauga, ON) to remove genomic DNA contamination. RNA quality was assessed using the Eukaryotic RNA 6000 Nano assay on a 2100 Bioanalyzer (Agilent, Mississauga, ON). Only samples with an RIN > 7 and a 28S:18S rRNA ratio >1.0 were used to prepare sequencing libraries. RNA sequencing libraries (one library per fish, three fish per treatment per species; total of 18 samples or libraries) were prepared and sequenced at the McGill University and Genome Quebec Innovation Centre (McGill University, Montreal, QC) using the TruSeq stranded mRNA library protocol and 100‐bp paired‐end sequencing in two lanes of an Illumina HiSeq 2000 sequencer (Illumina Inc., San Diego, CA).

Raw reads were pooled by species and de novo transcriptome assemblies were created for each species of goby using Trinity v3.0.3 (Grabherr et al., 2011). De novo assemblies were created using the default parameters and included a quality‐filtering step using default Trimmomatic v0.32 (Bolger, Lohse, & Usadel, 2014) and in‐silico normalization methods as implemented in Trinity. Raw reads for each sample were then individually quality filtered using Trimmomatic v0.32. Cleaned reads were multimapped to the reference transcriptome generated by Trinity for that species using Bowtie2 (Langmead & Salzberg, 2012) to report all valid mappings using the”—a” method. Further details of the specific parameters used for each software program are available in the Appendix S1 in the form of a custom unix shell script used to perform quality trimming and read mapping. Aligned reads for all samples of each species were processed using the program Corset v1.0.1 (Davidson & Oshlack, 2014), which uses information from the shared multimapping of sequence reads to hierarchically cluster the transcript contigs produced by de novo assembly into “genes” while using information about the treatment groups of individuals to split grouping of contigs when the relative expression difference between the contigs is not constant across treatments groups. Thus, Corset simultaneously clusters gene fragments generated during de novo assembly while separating paralogous genes and finally enumerates read counts for each of these genes (Davidson & Oshlack, 2014). This method performs as well or better than other current methods for clustering transcripts generated during de novo assembly (Davidson & Oshlack, 2014). To focus on biologically relevant transcriptional changes and avoid statistical issues for genes with low numbers of counts, we removed genes that did not meet a minimum expression level of at least one count per million reads in at least three samples (within one treatment) prior to analysis. To assess the consistency of our data and visually validate the use of three biological replicates per treatment, we conducted principal component analysis on centered and scaled count data as implemented in the “ade4” v1.7‐4 package (Dray & Dufour, 2007) in R v3.1.3 (R Core Team 2016) for each species individually and then the two species combined for putative orthologous genes.

To test the hypothesis that round goby have an increased capacity for transcriptional response, we conducted two sets of complimentary analyses. The first set of analyses focused on the quantification of the ability of gobies to alter transcriptome‐wide gene expression in response to environmental perturbation (temperature treatments). The second set of analyses focused on the function of responding genes, and whether genes with plastic responses to environmental perturbations represented relevant and coordinated biological functions for dealing with the temperature stress or random transcriptional changes lacking directed biological function.

#### 2.2.1 Transcriptome‐wide plasticity

We used univariate generalized linear models (GLM) to identify differentially expressed genes in response to each temperature challenge for each species of goby separately. Negative binomial GLMs were implemented using the “edgeR” v3.8.6 package (Robinson, McCarthy, & Smyth, 2010) in R v3.1.3 (R Core Team 2016) using a false discovery rate of 0.05 to correct *p*‐values for multiple comparisons (Benjamini & Hochberg, 1995). Briefly, the “edgeR” approach normalizes count data using trimmed mean of M‐values (Robinson & Oshlack, 2010) that accounts for differences in library size among individuals. Negative binomial models are then fitted to the normalized count data for individuals, gene by gene, using gene‐specific dispersion parameters estimated from the data using an empirical Bayes approach (McCarthy, Chen, & Smyth, 2012). Statistical significance of model terms is then tested using a likelihood ratio test. Genes identified as being differentially expressed in response to temperature represent gene transcription that is responding plastically to environmental cues.

To assess differences between round and tubenose goby for transcriptome‐wide scope (magnitude of transcriptional change) for response, we first compared the distribution of Log_2_ fold changes in transcription response to temperature challenges for all genes irrespective of statistical significance. We tested for differences in the rank order of fold change between species for upregulated (positive Log_2_ fold change) and downregulated (negative Log_2_ fold change) genes separately in each treatment using nonparametric Wilcoxon rank‐sum tests in R v3.1.3 (R Core Team 2016). This analysis provides an estimate of transcriptional variability not explicitly influenced by temperature. We then considered the specific difference between species in the scope of transcriptional response for genes that were identified as statistically significantly responding to temperature challenge. For this analysis, we considered only Log_2_ fold changes from the genes that were identified as being significantly differentially expressed individually by each species in the GLMs above. Nonparametric Wilcoxon rank‐sum tests were again used to compare the rank order of fold change between species for upregulated and downregulated genes separately in each treatment.

To further facilitate comparison of gene transcription variation between species and allow combining the species‐specific datasets, we identified putative orthologous genes using reciprocal best blast hits for round goby and tubenose goby transcripts using the blastn algorithm from BLAST+ v2.19 (Camacho et al., 2009). We retained valid putative orthologs only where both transcripts were each other's best matches. While this is a simple approach to identifying gene orthologs, it has been shown to outperform many more sophisticated algorithms (Altenhoff & Dessimoz, 2009). We recognize the need for further phylogenetic assessment to verify our putative gene pairs are in fact orthologs and not extra‐paralogs and so we refer to our orthologs throughout as “putative” to reinforce their preliminary designation. We used the putative orthologous gene information to analyze paired comparisons of species‐specific Log_2_ fold changes to temperature in each challenge (Log_2_ fold change from species‐specific one‐way GLMs above). We included only orthologous genes identified as statistically significantly responding to temperature challenge based on the two‐factor GLMs. Here, we analyzed the paired comparison of Log_2_ fold changes between the two species of goby for upregulated and downregulated genes separately in each treatment with Wilcoxon signed‐rank tests, a nonparametric analog of a paired *t*‐test.

We then combined the raw gene transcription count data from both species for genes that were putatively orthologous and tested for species differences in transcription at the shared expressed genes using two‐factor GLMs for each temperature challenge. The two‐factor negative binomial GLMs were implemented in “edgeR,” with gene‐specific dispersion parameters estimated as described above, using the following model:

(1) $$X_{ijk}\;=\;T_{i}+S_{j}+I_{ij}+e_{ijk}$$

where *T*_*i*_ represents the effect of temperature treatment (control versus treatment), *S*_*j*_ represents the effect of species, *I*_*ij*_ the species × temperature interaction, and *e*_*ijk*_ the residual error. Genes exhibiting a species‐by‐treatment interaction could reflect transcriptional response capacity possessed or utilized by one species but not the other and may thus be the basis of differential invasion success. Additionally, maintenance of biological function may be more transcriptionally demanding and the scope for response may be limited due to higher levels of constitutive transcription for genes in one species. To assess this, we identified orthologous genes that were statistically significantly differentially transcribed between species based on the likelihood ratio test for the species term from the two‐factor GLMs. We then used the Log_2_ fold change associated with statistically significant genes to assess the magnitude that one species over‐transcribed a gene relative to the other. In this context, positive fold changes indicated genes consistently transcribed higher by tubenose goby irrespective of temperature treatment and negative fold changes indicated genes consistently transcribed higher by round goby. Wilcoxon rank‐sum tests were used to test for a difference between round and tubenose goby in the magnitude of over transcription between the two species. For this analysis, we only considered genes significantly differently transcribed between species and not exhibiting an interaction effect.

### 2.3 Plasticity in gene function

The second set of analyses investigated differences in regulation of gene function between round and tubenose goby. We annotated our sequences with Gene Ontology (GO; Ashburner et al., 2000) information using Blast2GO v3.1 (Conesa et al., 2005). Briefly, transcript sequences were compared for sequence homology to records in the nonredundant (nr) protein database of the National Center for Biotechnology Information (http://www.ncbi.nlm.nih.gov) using the blastx algorithm from BLAST+ v2.19 (Camacho et al., 2009) with an e‐value cutoff of 0.001. Goby transcripts were then associated with GO terms based on the GO annotations for the transcripts' top BLAST hits using the GO association database from 15 September 2015 (The Gene Ontology Consortium, 2015). To account for transcript length biases in the ability to detect differential expression from RNAseq data, we tested for over‐representation of GO categories present in our contrasts of interest using the “goseq” v1.18 package (Young, Wakefield, Smyth, & Oshlack, 2010) in R v3.1.3 (R Core Team 2016). Specifically, we tested for functional enrichment (over‐representation) for all GO categories represented by a minimum of five annotated genes. We tested up‐ and downregulation of biological processes to increased or decreased temperature relative to all genes with annotation for each species separately. We corrected for multiple comparisons using a false discovery rate of 0.05 (Benjamini & Hochberg, 1995). Additionally, we identified the genes that exhibited the strongest response to temperature challenge for each species (top 5% of fold increase or decrease in transcription in each temperature treatment). We tested for functional enrichment of GO biological processes represented by those genes in the same manner as above to discover the most plastic functions in each species that might be important for explaining the difference in performance between them.

## 3 Results

### 3.1 RNA sequencing and de novo transcriptome assembly

**Figure 2:**
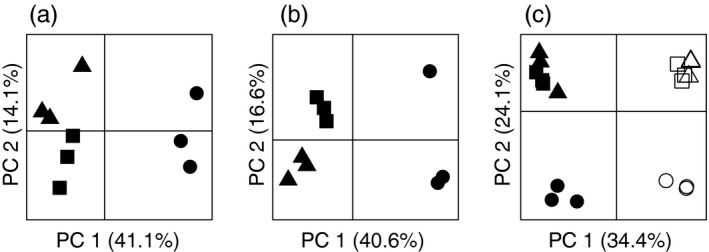
Principle component bi‐plots of the first two principle components derived from gene transcription count data between samples for all genes for round goby (a), tubenose goby (b), and putative orthologous genes for both species combined (c) from three acute temperature treatments: control—18°C (squares), cold treatment—5°C (circles), and warm treatment—25°C (triangles). Round goby are represented by the solid symbols and tubenose goby by the open symbols in panel c

We generated 214.9 million 100‐bp paired‐end reads for round goby and 214.2 million 100‐bp paired‐end reads for tubenose goby with an even distribution of data among samples (Table S1). The Trinity assembly software reconstructed 213,329 transcript clusters for round goby and 188,405 transcript clusters for tubenose goby. Quality filtering of individual sample read sets using Trimmomatic retained 93%–95% of read pairs (Table S1). Of these, a large proportion of high‐quality read pairs (91%–94%) were mapped to the respective species de novo transcript reference (Table S1). Corset transcript clustering reduced the number of unique “genes,” or transcript clusters, to 63,231 for round goby and 57,468 for tubenose goby, and of these, 26,215 genes for round goby and 23,648 genes for tubenose goby were retained following filtering for minimum expression level (>1 count per million reads, e.g., approximately 20–25 reads across at least three fish). Principal component (PC) bi‐plots of the two largest PCs indicate good consistency among samples from each treatment (Figure 2). The first PC axis for both species describes approximately 40% of the transcriptional variation and is driven by the difference in expression of the cold treatment and likely reflects the magnitude of temperature change for the cold treatment relative to the warm treatment. The second PC axis for both species explains approximately 15% of the transcriptional variation and generally separates the warm treatment from the control treatment (Figure 2), although it does capture some within‐group variation especially for the cold treatment tubenose goby (Figure 2b). This within‐group variation is unlikely to be due to age differences and all fish appeared to be in good condition prior to experimentation; however, it could reflect a sex difference, as we were unable to obtain sex information for these fish. The PCA combining round and tubenose goby for the putative orthologous genes identified similar patterns; however, species differences appear to explain as much or more of variance in transcription than the temperature challenge (Figure 2c).

### 3.2 Transcriptome‐wide plasticity

**Figure 3:**
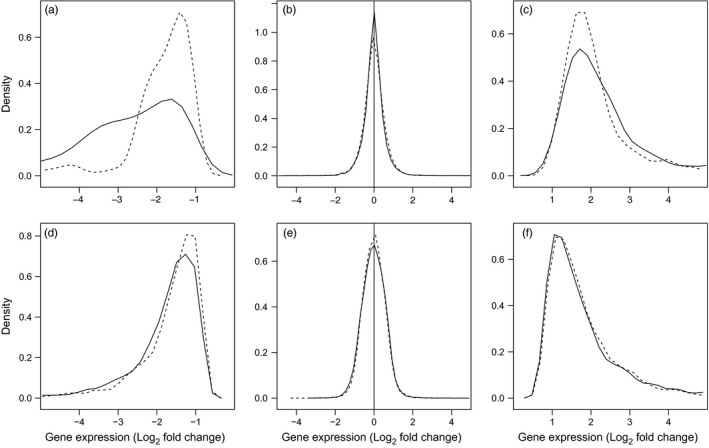
Differences between round and tubenose goby in the distribution of Log_2_ fold changes of gene transcription in response to increased temperature challenge (a–c) and decreased temperature challenge (d–f). Lines represent the relative density (amount) of genes corresponding to the fold change indicated on the x‐axis for round goby (solid lines) and tubenose goby (dashed lines). Panels present genes with statistically significant downregulation of transcription (a, d), no transcriptional plasticity (b, e), and statistically significant upregulation of transcription (c, f) as determined for each species using negative binomial generalized linear models (FDR < 0.05, see Section 2). The generally higher density of genes for tubenose goby at lower magnitude fold changes indicates reduced scope for transcriptional plasticity. The shift of the distribution between species is statistically significant for comparisons a, c, and d based on Wilcoxon rank‐sum tests (Table 1)

**Figure 4:**
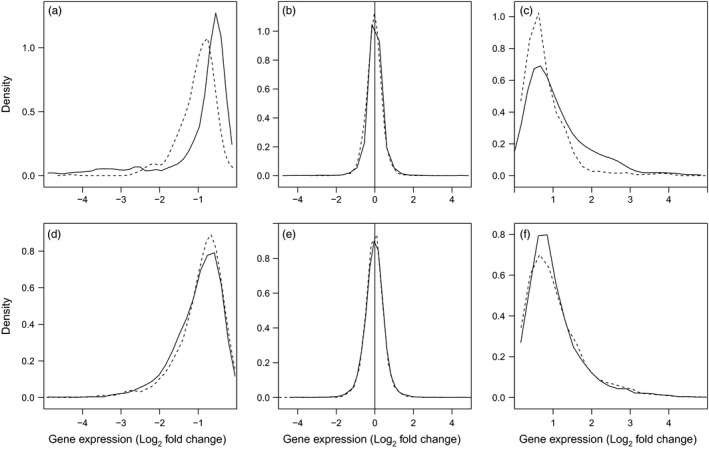
Differences between round and tubenose goby in the distribution of Log_2_ fold changes of transcription for identified putative orthologous genes in response to increased temperature challenge (a–c) and decreased temperature challenge (d–f). Lines represent the relative density (amount) of genes corresponding to the fold change indicated on the x‐axis for round goby (solid lines) and tubenose goby (dashed lines). Panels present genes with statistically significant downregulation of transcription (a, d), no transcriptional plasticity (b, e), and statistically significant upregulation of transcription (c, f) as determined for each species using negative binomial generalized linear models (FDR < 0.05, see Section 2). The generally higher density of genes for tubenose goby at lower magnitude fold changes indicates reduced scope for transcriptional plasticity. The shift of the distribution between species is statistically significant for comparisons a, c, and d based on Wilcoxon rank‐sum tests (Table 1)

To first characterize transcriptome‐wide patterns of plasticity, we identified differentially expressed genes using univariate GLMs for each species and temperature treatment. Results from the individual species GLMs indicate that only a minority of genes in both species responded plastically to temperature challenge (high temperature: ~2%; low temperature: ~22%; Table 1). The patterns of differential transcription in terms of the proportions of differentially expressed genes are similar between the two species (Table 1). In contrast, Log_2_ fold changes were on average greater in magnitude for round goby in all comparisons except for genes upregulated in response to cold, where there was no significant difference (Table 1; Figure 3). This indicates that round goby have an increased scope for transcriptional plasticity compared with tubenose goby. When considering only the putative orthologous genes, the pattern remains the same, except for genes downregulated in response to high temperature where the pattern of greater average fold change is higher for tubenose goby (Table 1; Figure 4).

**Table 1:** Gene transcriptional response of all genes and for paired putative orthologous genes from round and tubenose goby exposed to cold and hot temperature challenges (*N*: number of genes in category for RG: round goby or TNG: tubenose goby, mean (*SD*): average (standard deviation) of Log_2_ fold change in response to temperature challenge, Wilcoxon W: W statistic for Wilcoxon test, *p* value: *p*‐value for Wilcoxon test)

	RG		TNG		Wilcoxon	*p* value
	*N*	Mean (*SD*)	*N*	Mean (*SD*)	W	
All genes						
Increased temperature	26,215	0.423 (0.58)	23,648	0.417 (0.46)	2.96 × 10^8^	<2.2 × 10^−16^
Decreased temperature	26,215	0.771 (0.82)	23,648	0.726 (0.77)	3.20 × 10^8^	9.6 × 10^−11^
Differentially expressed genes						
Increased temperature						
Upregulated	308	2.55 (1.50)	225	2.29 (1.32)	3.85 × 10^4^	.029
Downregulated	334	−2.83 (1.56)	199	−2.01 (1.24)	4.64 × 10^4^	1.6 × 10^−14^
Not DE	25,573		23,224			
Decreased temperature						
Upregulated	2,922	1.84 (1.09)	2,806	1.83 (1.04)	4.02 × 10^6^	.21
Downregulated	2,941	−1.80 (0.99)	2,264	−1.67 (0.91)	3.68 × 10^6^	1.1 × 10^−10^
Not DE	20,352		18,578			
Orthologous genes						
Increased temperature						
Upregulated	345	1.11 (0.90)	345	0.75 (0.81)	3.9 × 10^4^	4.6 × 10^−7^
Downregulated	338	−0.98 (0.99)	338	−1.01 (0.49)	2.1 × 10^4^	2.1 × 10^−5^
Not DE	10,481		10,481			
Decreased temperature						
Upregulated	2,313	0.99 (0.77)	2,313	1.00 (0.78)	1.4 × 10^6^	.70
Downregulated	2,418	−1.01 (0.67)	2,418	−0.93 (0.60)	1.59 × 10^6^	6.9 × 10^−5^
Not DE	6,433		6,433			

The two‐factor GLMs with species and temperature as factors identified 76 (0.7%) gene orthologs with a significant species‐by‐temperature interaction effect in the high‐temperature treatment and 823 (7.3%) gene orthologs in the cold temperature treatment. Functional annotation was available for 44 gene orthologs demonstrating a significant interaction in the high‐temperature treatment and 560 gene orthologs in the cold temperature treatment. The only biological process significantly over‐represented by any of these responses was present in response to cold temperature challenge and was for genes involved in steroid hormone‐mediated signaling (GO: 0043401, 11 differentially expressed genes, 35 total genes with this GO annotation, FDR = 0.0097, Fig. S1). These genes, and the other genes demonstrating an interaction between species and temperature challenge (Table S2), may represent the transcriptomic basis of the differential performance of these species and are candidates for further study.

**Figure 5:**
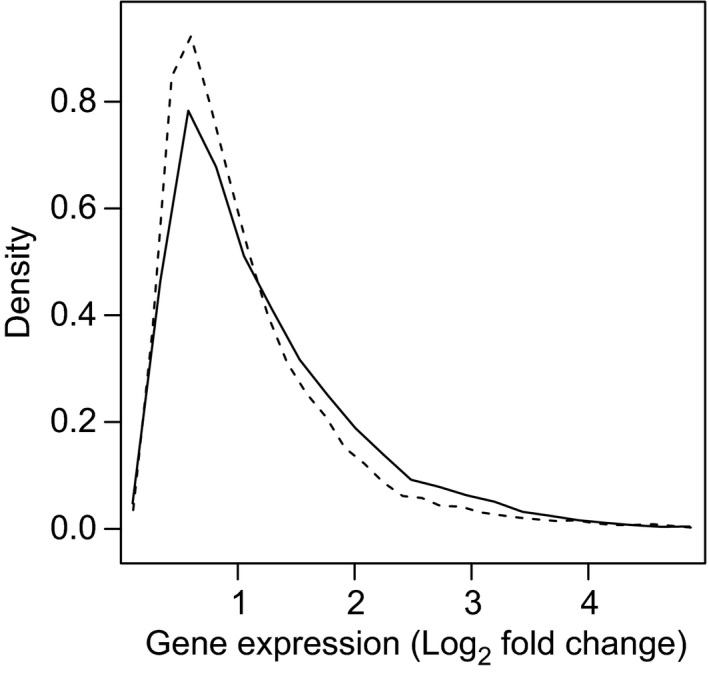
Distribution of Log_2_ fold changes of transcription for putative orthologous genes differentially transcribed (FDR < 0.05) between round and tubenose goby. Lines represent the relative density (amount) of genes corresponding to the magnitude of fold change indicated on the x‐axis for orthologous genes one species over‐transcribes relative to the other. Genes transcribed higher in round goby are represented by the solid lines and genes transcribed higher in tubenose goby are represented by the dashed lines. Tubenose goby over‐transcribes genes to a greater magnitude than round goby based on a Wilcoxon rank‐sum test (*p* < .0001)

Of the 10,265 putative orthologs not exhibiting an interaction effect between species in either treatment, 6,782 (66.1%) of them are significantly differently transcribed between the two species. These represent 3,346 genes (49.3%) transcribed at a higher level in tubenose goby (mean Log_2_ fold difference: 1.23) and 3,441 genes (50.7%) transcribed at a higher level in round goby (mean Log_2_ fold difference: 1.08). There is a significant difference in the magnitude of differential transcription between goby species (W = 6.04 × 10^6^, *p* = 1.8 × 10^−15^). The genes that tubenose goby over‐transcribes relative to round goby are over‐transcribed to a greater degree than the genes that round goby over‐transcribes relative to tubenose goby (Figure 5). This difference corresponds to tubenose goby having, on average, 11% higher transcription of orthologous genes compared to round goby. This pattern of higher average transcription in tubenose goby is largely driven by differences in constitutive expression of genes not responding plastically to temperature challenge (Table 2), although there is a significant difference in the magnitude of transcription between species for genes upregulated in response to decreased temperature.

**Table 2:** Magnitude of Log_2_ fold difference between round and tubenose gobies for genes plastically responding to increased or decreased temperature and those not responding to temperature (*N*: number of genes in category higher for RG: round goby or TNG: tubenose goby, mean (*SD*): average (standard deviation) of Log_2_ fold increase over the other species, Wilcoxon W: W statistic for Wilcoxon rank‐sum test for rank order of RG versus TNG for that category of genes, *p* value: *p*‐value for Wilcoxon rank‐sum test)

	RG		TNG		Wilcoxon	*p* value
	*N*	Mean (*SD*)	*N*	Mean (*SD*)	W	
Increased temperature						
Upregulated	51	1.33 (0.90)	92	1.37 (0.87)	2.25 × 10^3^	.712
Downregulated	95	1.02 (0.85)	43	1.16 (0.77)	1.78 × 10^3^	.232
Decreased temperature						
Upregulated	639	1.01 (0.75)	538	1.18 (0.78)	1.43 × 10^5^	8.28 × 10^−7^
Downregulated	693	1.02 (0.67)	700	1.13 (0.71)	2.18 × 10^5^	.001
No temperature response						
No difference	1,806	1.11 (0.83)	1,825	1.27 (0.94)	1.48 × 10^6^	1.43 × 10^−7^

### 3.3 Plasticity in gene function

The second set of analyses investigated biological function associated with transcriptional changes in response to temperature challenge. Functional annotation was possible for 10,777 genes in round goby and 10,695 genes in tubenose goby. We characterized biological process categories in the Gene Ontology framework that were over‐represented by genes either up‐ or downregulated in response to increased and decreased temperature for each species separately.

**Figure 6:**
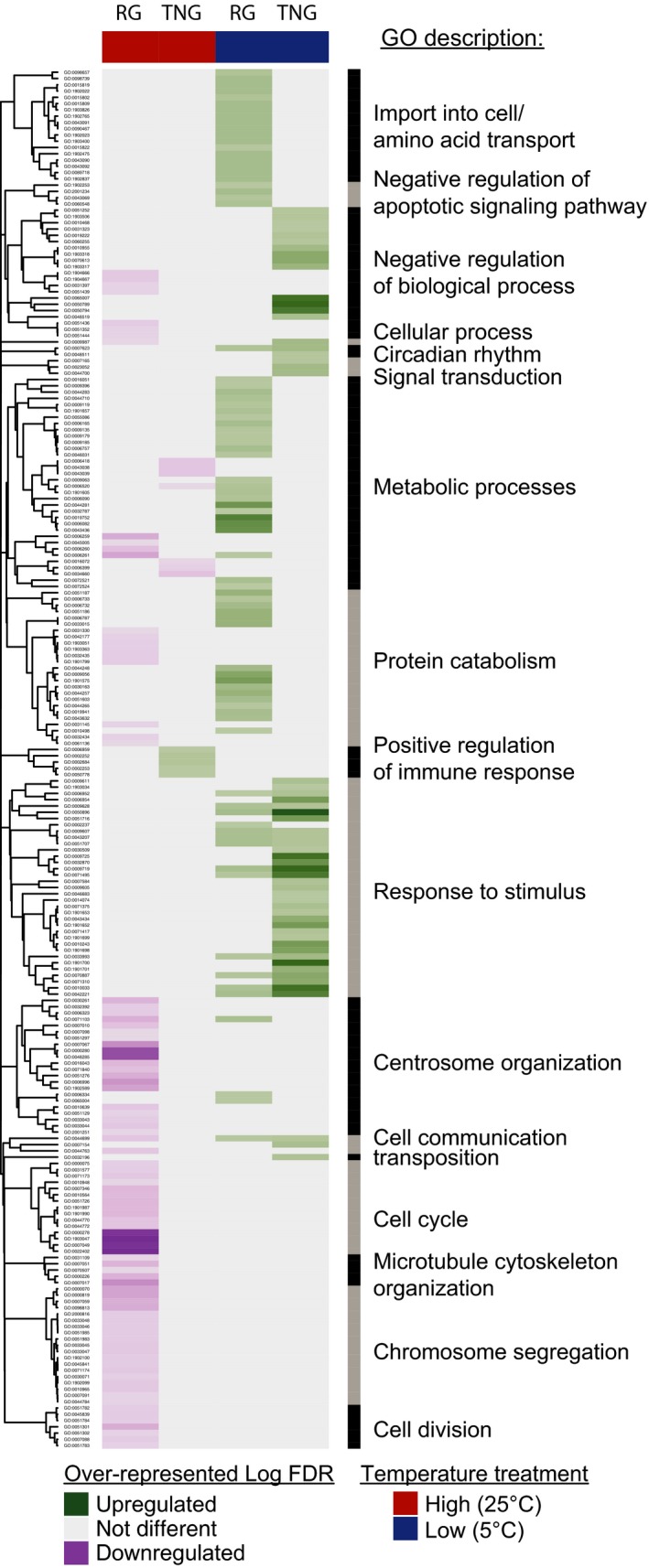
Heatmap of gene ontology (GO) biological process categories over‐represented by genes either upregulated (green) or downregulated (purple) in round goby (RG) and tubenose goby (TNG) liver tissue in response to two acute thermal challenges. GO biological process over‐representation tests were performed using the “goseq” v1.18 package in R v3.1.3 (R Core Team 2016; Young et al., 2010). Statistically significant processes after false discovery rate correction (Benjamini & Hochberg, 1995) were grouped across species and treatment and clustered based on semantic similarity criterion of Schlicker, Domingues, Rahnenführer, and Lengauer (2006) as implemented in the “GOSemSim” v1.99.4 package (Yu et al., 2010) in R and “complete” hierarchical clustering as implemented in the “hclust” function in R. Full GO over‐representation results are available in Table S3

Round goby did not exhibit over‐representation of upregulated transcription for any biological processes in response to increased temperature but did exhibit over‐representation of downregulation for a variety of biological processes (*N* = 89), most of which were related to cell cycle, DNA replication, and cell division (Figure 6, Table S3). The round goby also exhibited over‐representation of downregulated genes involved in the repression of ubiquitin‐mediated proteolysis, which should result in the upregulation of this function. In contrast, tubenose goby exhibited over‐representation of upregulated transcription of five biological processes, all involved in humoral immunity and activation of the immune response. Tubenose goby exhibited over‐representation of downregulated transcription of biological processes (*N* = 7) mostly involved in rRNA and tRNA metabolic processes and tRNA activation (Figure 6, Table S3) suggesting a general reduction in gene translational activity in response to increased temperature.

In response to decreased temperature, round goby exhibited over‐representation of many upregulated biological processes (*N* = 81), including carboxylic acid metabolic processes typical of phospholipid membrane alterations, transport of basic amino acids (arginine and lysine), and biosynthesis of carbohydrates typical of antifreeze functions, negative regulation of apoptosis, and proteosomal activity characteristic of targeted degradation or turnover of proteins (Figure 6, Table S3). Tubenose goby also exhibited over‐representation of many upregulated biological processes (*N* = 57) in response to decreased temperature, but with very different functional implications. The majority of upregulated processes were response to stimulus processes indicative of detection of stimulus, cell signaling cascades, regulation of gene expression, and immune system processes (Figure 6, Table S3). Neither species of goby exhibited any over‐representation of downregulated biological processes in response to reduced temperature, after correction for multiple tests. Interestingly, round and tubenose goby shared 14 biological processes that were over‐represented by genes upregulated in response to decreased temperature (Figure 6, Table S3). All of these processes were for response to stimulus suggesting that these species were both able to detect the changes in their environment and produce signaling cascades to direct biological functions as a result. The lack of many other processes regulated by tubenose goby could suggest either they lack specific mechanisms to deal with the stress they experienced or that there may be a difference in the timing of the onset of the response.

To characterize the most plastic biological functions for each species in response to temperature challenge, we identified genes with the largest Log_2_ fold changes (top 5%) within the significantly up‐ and downregulated genes separately in each temperature treatment (Table 3). Significantly over‐represented biological processes represented by these highly plastic genes were only evident for upregulated genes in response to the cold temperature treatment for both species. Round goby demonstrated over‐representation of 28 biological processes, whereas tubenose goby only demonstrated over‐representation of five biological processes (Table S4). Two processes were shared between both species relating to alcohol and polyol biosynthesis that may be related to antifreeze capacity and cold tolerance. Round goby exhibited extreme plasticity for additional processes related to oxygen binding and carbohydrate metabolism, while tubenose goby exhibited plasticity for ceramide metabolic process potentially related to signaling cellular stress.

**Table 3:** Magnitudes of most plastic gene transcription (top 5% of Log_2_ fold change) for round goby (RG) and tubenose goby (TNG) in response to acute temperature challenge. *N* = number of genes in top 5% of fold change, R = range of Log_2_ fold changes for genes

	RG		TNG	
	*N*	R	*N*	R
Increased temperature				
Upregulated	6	4.1–8.2	4	3.9–10.1
Downregulated	8	5.2–8.1	6	2.6–8.3
Decreased temperature				
Upregulated	67	3.1–8.1	60	3.1–9.5
Downregulated	56	3.1–7.8	50	2.8–7.4

## 4 Discussion

We demonstrated liver tissue transcriptional differences between round and tubenose gobies in response to acute temperature challenges that may contribute to the dramatic differences in the geographic extent of invasion of these two species. Round goby possessed a greater scope for transcriptional response to altered temperature compared with tubenose goby. The two species exhibited a similar number of genes with significantly altered transcriptional state; however, the transcriptional changes by tubenose goby failed to represent the same biological processes altered by round goby. Furthermore, the functions of the genes that responded to the challenges in round goby, but did not in tubenose goby, were consistent with adaptive responses to maintain or regain homeostasis following rapid changes in temperature. The capacity for transcriptional plasticity to environmental stressors has potential as an important predictor of the physiological tolerances of organisms (López‐Maury et al., 2008; Whitehead, 2012). Physiological tolerances ultimately define species' distributions, capacity for range expansion, and, therefore, potential for invasion success.

The response of round goby to thermal stress suggests that it can transcriptionally respond to maintain biological function over a broader range of temperatures than tubenose goby. This result is consistent with round goby having a higher thermal limit than tubenose goby (Xin, 2016). Given the more dramatic differences we observed in transcriptional response to cold treatment between species and the role of cold tolerance in determining invasion success in the Great Lakes (Kolar & Lodge, 2002), we suggest further investigation into the thermal performance curves for tubenose goby and determination of lower thermal limits for these species would be worthwhile. Broad thermal tolerance has been previously associated with higher invasion success (Bates et al., 2013), and our transcriptional results suggest that capacity for transcriptional response is a potential mechanism that explains the differential invasion success between goby species in our study.

Reduced scope of gene transcription response to specific environmental challenges (in our case, temperature) implies a reduced capacity to acclimate to a broad range of environments and may have limited the range expansion of tubenose goby. Indeed, Antarctic fishes that have evolved in very stable environments have completely lost a heat shock response (for a review, see: Logan & Buckley, 2015). Reduced transcriptional capacity to respond to heat stress has also been documented for fish species that only have a moderate temperature tolerance range (*Hypomesus transpacificus*, Komoroske, Connon, Jeffries, & Fangue, 2015) compared to the transcriptional responses of fish species that are known to tolerate a broader range of temperatures (e.g., *Gillichthys miribilis*, Logan & Somero, 2011). The evolution of plasticity is thought to be constrained by the relative cost of having a plastic phenotype compared with exhibiting a canalized phenotype (Agrawal, 2001). It is possible that tubenose goby have experienced a greater cost to being transcriptionally plastic in its native range than round goby that resulted in the evolution of a reduced transcriptional response to acute thermal challenge; however, we cannot rule out genetic drift as a mechanism explaining the difference either (Whitehead, 2012). Alternatively, increased transcriptional response may not always be indicative of tolerance; for example, if a stressor is mild, a highly tolerant species may not respond transcriptionally at all, and there are examples of pollutant tolerant fish that have evolved a muted transcriptional response to pollution exposure (Whitehead, Triant, Champlin, & Nacci, 2010). In our case, the combination of the species‐level performance (invasion range expansion and impact) and physiological differences (thermal limits and metabolic rates) makes it unlikely that tubenose goby were able to maintain homeostasis despite a reduced transcriptional responses to temperature challenge.

In addition to increased capacity for transcriptional plasticity, the transcriptional changes exhibited by round goby are more consistent with adaptive responses to thermal challenge than those observed in the tubenose goby. Round goby altered biological processes that are characteristic of acute responses to temperature reported in other species with broad thermal tolerance (e.g., ubiquitin‐dependent protein degradation and negative regulation of apoptosis; Logan & Somero, 2011) and are believed to help organisms survive and recover from acute stress events (Wiseman et al., 2007). In contrast, tubenose goby responded to the challenge by altering a similar number of genes; however, with the exception of innate immune response to tissue damage, tubenose goby did not respond with the same biological processes as round goby. This highlights an important difference between adaptive and maladaptive phenotypic plasticity. That is, phenotypic plasticity is only beneficial for an organism when it alters phenotype (partially or fully) in the direction of a peak on a fitness landscape (increases fitness; Ghalambor et al., 2007). If plasticity alters a phenotype in a direction other than toward a fitness peak, as it does for tubenose goby where a similar number of transcriptional changes as round goby do not represent a similar functional response, these plastic changes may result in no or even negative fitness consequences for the organism. Variation in the timing of transcriptional response to a stressor (e.g., Whitehead et al., 2012) could explain the observed difference between species; however, delayed induction of biological responses by tubenose goby would likely also be maladaptive, especially if it resulted in delayed compensatory responses that are necessary for short‐term survival.

The reduced scope of transcriptional response of tubenose goby suggests either that it lacked the biological mechanisms to respond to acute thermal stress or that tubenose goby found the handling procedures stressful and thus suffered reduced capacity to respond to the heat stress. While we could have conducted a laboratory acclimation experiment to isolate temperature as the sole factor driving transcriptional changes in our gobies, temperature is not the only environmental stressor encountered by these organisms. We provide a comparison of transcriptional response to temperature stressors that reflects the organisms' ecological context while controlling for prior environmental exposure by sampling these organisms from the same habitat at the same time. Presumably, sensitivity to the synergistic effects of multiple stressors expressed as a reduction in a potential aquatic invader's transcriptional capacity would not be adaptive for the invading species. Our use of three biological replicates has the potential to result in inflated variance estimates that inhibit our ability to detect more subtle differential expression; thus, our list of differentially transcribed genes should be considered conservative. Despite this limitation, we have characterized hundreds to thousands of differentially transcribed genes in each treatment (Table 1) and our treatments are well separated in multivariate space suggesting within‐group error is not a limiting factor (Figure 2). The proportions of differentially responding genes we report are comparable to other studies of acute thermal stress (Logan & Somero, 2011; Quinn et al., 2011) suggesting that despite the lack of laboratory acclimation, we still captured important biological responses in an ecological context.

The process of invasion or range expansion often results in genetic founder effects and bottlenecks (Dlugosch & Parker, 2008) and the resulting reductions in genetic diversity have potential consequences for adaptive capacity. Phenotypic plasticity, when adaptive, is widely believed to help buffer species from the selective forces of novel environments (Ghalambor et al., 2007; Lande, 2015); however, plasticity itself can evolve. The evolution of increased plasticity is expected to be favored early in the process of invasion, while selection in the invaded range is expected to eventually reduce plasticity (Lande, 2015). One of the key issues regarding empirical assessment of the role of plasticity in invasions is controlling for the time since invasion (Lande, 2015). The goby species presented here have similar invasion histories (both first detected in St. Clair River in 1990, Jude et al., 1992) and have similar ages at maturity (females at age 1; round goby: MacInnis & Corkum, 2000a; tubenose goby: Valová et al., 2015) indicating that a similar number of generations since invasion have occurred for both species. It is therefore unlikely that tubenose goby has had enough time to evolve a loss of plasticity in North America, while the round goby has not. Alternatively, the stochastic processes associated with founder effects may have prevented tubenose gobies bearing the full range of plastic phenotypes in the native range from becoming established in the first place. There is no evidence that tubenose goby have experienced greater founder or bottleneck effects during their North American invasion than round goby (Stepien & Tumeo, 2006) making differences in genetic diversity an unlikely explanation for the observed differences in transcriptional plasticity.

The lower transcriptional plasticity we found in the tubenose goby may reflect source population characteristics if selection pressures among assemblages of tubenose goby in their native range resulted in local adaptation, while the round goby in their native range are one broadly tolerant species. Round goby is known to exhibit broad environmental tolerance to other abiotic stressors, including salinity (Karsiotis, Pierce, Brown, & Stepien, 2012) and contaminants (McCallum et al., 2014). While less is known about the specific physiological tolerances of tubenose goby, the two species are found in similar habitats in both their native (Kottelat & Freyhof, 2007) and invaded ranges (Jude & DeBoe, 1996) suggesting they have evolved under similar conditions for at least the past several thousand years. The phylogeny of tubenose goby in the northern Black Sea is represented by multiple divergent lineages (Neilson & Stepien, 2009; Sorokin, Medvedev, Vasil'ev, & Vasil'eva, 2011) only one of which has invaded North America (Neilson & Stepien, 2009). In contrast, round goby from this same region form one monophyletic group (Brown & Stepien, 2008).

There has been a tendency for invasion biologists to treat organisms as static entities and ignore the role of plasticity and evolution in determining invasion risk (Whitney & Gabler, 2008). Plasticity may confer invasion success by either increasing fitness in both unfavorable and favorable environments (Richards, Bossdorf, Muth, Gurevitch, & Pigliucci, 2006). Broad thermal tolerance should increase fitness in unfavorable environments and has been associated with range expansions (Bates et al., 2013). The role of transcriptional plasticity in determining thermal tolerance suggests that assessment of transcriptional profiles under thermal stress may be a valuable tool to assess invasion risk. Our results demonstrate the power of using measures of transcriptional variation to detect meaningful biological responses to thermal stress in an ecological context that would be directly relevant to a species' ability to survive, uptake transport, and establishment in a novel environment. Comparative genomics has enormous potential to identify the mechanistic basis of variable acclimation capacity among groups of organisms (Whitehead, 2012). We have used a comparative approach to further demonstrate that differences in transcriptional response to acute temperature challenge may underlie the difference in invasion success between our two study species. Conservation biologists have embraced the use of transcriptomic profiles to identify and select more plastic source populations to maximize the success of species reintroductions (He, Johansson, & Heath, 2016). Managing invasive species is simply applying this approach in reverse, where managers would want to prioritize prevention of transport and establishment of the most plastic invaders. Assessing transcriptional plasticity in response to acute stressors, such as temperature, combined with knowledge of the relationship between transcription and physiology (e.g., high transcriptional response is beneficial for thermal acclimation but may be maladaptive for pollution tolerance) would provide managers with objective measures of the plastic capacity of potential invasive species. Such data are critical for effective invasion risk assessment and the incorporation of quantitative approaches into invasion risk assessment will change how invasive species are managed and their impacts minimized.

## Data Accessibility

Raw sequencing data for both species are available at the NCBI Sequence Read Archive under project accession numbers SRP075124 and SRP075141. Scripts used to process raw data, assemble the transcriptome, and generate the count data file as well as the count data file and R scripts used to perform the differential expression analysis are available on Dryad: https://doi.org/10.5061/dryad.408ht.
