# Supplementary material for: Plasticity in gene transcription explains the differential performance of two invasive fish species
Source: Evol Appl. 2017 Apr 25;10(6):563–76. doi: 10.1111/eva.12463 (PMC5469171; doi:10.1111/eva.12463)
Supplement: Supplementary file 1 [file EVA-10-563-s001.docx]

Supplemental Material:

Table S1: RNA sequencing and read mapping summary statistics for round and tubenose goby. Read mapping statistics refer to mapping of the sample with bowtie2 to the respective species-specific *de novo* assembled transcriptomes.

| Sample Name | Number of Reads | Number of Bases | Average Quality | % Duplicate | Reads pairs passing QC | Read pairs mapping once | Read pairs mapping more than once |
| --- | --- | --- | --- | --- | --- | --- | --- |
| **Tubenose goby** | |  |  |  |  |  |  |
| L04 | 2.58E+07 | 5.16E+09 | 35 | 40.971 | 2.46E+07 | 3.65E+06 | 1.95E+07 |
| L03 | 2.41E+07 | 4.83E+09 | 35 | 40.418 | 2.29E+07 | 3.31E+06 | 1.83E+07 |
| L02 | 2.29E+07 | 4.57E+09 | 35 | 36.974 | 2.17E+07 | 3.38E+06 | 1.70E+07 |
| H04 | 2.38E+07 | 4.76E+09 | 35 | 36.203 | 2.24E+07 | 3.48E+06 | 1.74E+07 |
| H02 | 2.35E+07 | 4.70E+09 | 35 | 37.81 | 2.23E+07 | 3.40E+06 | 1.75E+07 |
| H01 | 2.22E+07 | 4.43E+09 | 35 | 34.87 | 2.09E+07 | 3.19E+06 | 1.64E+07 |
| C04 | 2.44E+07 | 4.87E+09 | 35 | 36.23 | 2.32E+07 | 3.62E+06 | 1.82E+07 |
| C03 | 2.32E+07 | 4.65E+09 | 35 | 38.046 | 2.20E+07 | 3.41E+06 | 1.73E+07 |
| C02 | 2.50E+07 | 5.01E+09 | 35 | 37.935 | 2.34E+07 | 3.39E+06 | 1.84E+07 |
| *Total* | *2.15E+08* | *4.30E+10* |  |  |  |  |  |
|  | |  |  |  |  |  |  |
| **Round goby** | |  |  |  |  |  |  |
| C02 | 2.40E+07 | 4.81E+09 | 35 | 34.535 | 2.24E+07 | 4.07E+06 | 1.63E+07 |
| C03 | 2.54E+07 | 5.08E+09 | 35 | 36.03 | 2.35E+07 | 4.45E+06 | 1.69E+07 |
| L01 | 2.16E+07 | 4.31E+09 | 35 | 34.253 | 2.01E+07 | 3.63E+06 | 1.47E+07 |
| L02 | 2.46E+07 | 4.93E+09 | 35 | 34.946 | 2.28E+07 | 4.16E+06 | 1.66E+07 |
| L03 | 2.53E+07 | 5.05E+09 | 35 | 37.027 | 2.34E+07 | 4.40E+06 | 1.69E+07 |
| C04 | 2.46E+07 | 4.92E+09 | 35 | 36.129 | 2.30E+07 | 4.13E+06 | 1.68E+07 |
| H01 | 2.27E+07 | 4.54E+09 | 35 | 35.434 | 2.15E+07 | 4.25E+06 | 1.55E+07 |
| H03 | 2.28E+07 | 4.55E+09 | 35 | 32.718 | 2.14E+07 | 4.19E+06 | 1.54E+07 |
| H04 | 2.32E+07 | 4.64E+09 | 35 | 32.846 | 2.19E+07 | 4.33E+06 | 1.58E+07 |
| *Total* | *2.14E+08* | *4.28E+10* |  |  |  |  |  |

Figure S1: Reaction norms for genes involved in the biological process of steroid hormone mediated signaling (GO:0043401) that demonstrated a significant interaction between species for response to cold temperature exposure.

Table S2: Annotated genes demonstrating an interaction between species and response to temperature challenge. RG = Round goby, TNG = Tubenose goby, LogFC = species specific Log_2_ fold change in response to high or low temperature challenge (bold values indicate statistical significance at FDR < 0.05) and gene IDs refer to the assembled transcripts for each species available on Dryad: XXXX.

|  | **Gene Name** | **RG LogFC** | **TNG LogFC** | **RG gene ID** | **TNG gene ID** |
| --- | --- | --- | --- | --- | --- |
| *High Temperature Challenge* | |  |  |  |  |
|  | cytochrome c oxidase subunit mitochondrial | -0.78 | **1.57** | Cluster-12194.0 | Cluster-9309.0 |
|  | antizyme inhibitor 1-like | 0.95 | -0.51 | Cluster-12916.1 | Cluster-28903.0 |
|  | c-c motif chemokine 20-like | **-0.42** | 3.90 | Cluster-15579.0 | Cluster-16837.0 |
|  | growth arrest and dna damage-inducible protein gadd45 alpha-like | **0.89** | -1.62 | Cluster-17200.0 | Cluster-9886.0 |
|  | solute carrier family 23 member 1 | **-2.27** | 0.72 | Cluster-19130.0 | Cluster-1785.7830 |
|  | collagen alpha-1 chain-like | -4.22 | **0.80** | Cluster-19832.0 | Cluster-15883.0 |
|  | splicing factor 3b subunit 2 isoform x2 | -0.28 | -1.56 | Cluster-21305.0 | Cluster-28631.0 |
|  | class e basic helix-loop-helix protein 41 | 0.62 | -1.74 | Cluster-22485.0 | Cluster-21169.0 |
|  | forkhead box protein o1-a-like | -0.94 | **0.39** | Cluster-23671.2 | Cluster-27976.0 |
|  | traf3-interacting protein 1 isoform x2 | -0.98 | **0.96** | Cluster-23777.1 | Cluster-16286.1 |
|  | mast cell protease 3-like | **1.40** | -1.01 | Cluster-24092.0 | Cluster-15286.0 |
|  | phenylalanine--trna ligase beta subunit | **0.26** | -1.39 | Cluster-24759.2 | Cluster-1785.12932 |
|  | erythrocyte band 7 integral membrane | **2.01** | -0.11 | Cluster-28795.0 | Cluster-15918.0 |
|  | deoxyribonuclease-1-like 2 | **-1.26** | 0.69 | Cluster-29355.0 | Cluster-1785.12596 |
|  | cd151 antigen-like | 0.47 | -1.08 | Cluster-29884.0 | Cluster-14117.0 |
|  | granulocyte colony-stimulating factor receptor | **2.47** | 0.19 | Cluster-30338.0 | Cluster-1785.12697 |
|  | zinc finger protein dzip1 isoform x1 | -3.68 | **0.02** | Cluster-30375.0 | Cluster-24831.0 |
|  | receptor-type tyrosine-protein phosphatase gamma | **-0.76** | 0.80 | Cluster-31819.0 | Cluster-10325.0 |
|  | atp-binding cassette sub-family f member 2 | **-0.62** | -2.62 | Cluster-33215.0 | Cluster-25534.0 |
|  | mitochondrial ornithine transporter 1-like | **0.64** | -1.79 | Cluster-33625.1 | Cluster-12977.0 |
|  | chromosome-associated kinesin kif4a | **-2.17** | 0.73 | Cluster-34189.2 | Cluster-1785.11754 |
|  | ras-related protein rab-33b-like | 2.36 | 0.01 | Cluster-34304.0 | Cluster-13260.0 |
|  | secretagogin-like | **-0.56** | **3.94** | Cluster-34969.0 | Cluster-1785.2823 |
|  | band 3 anion transport protein | -1.58 | **-0.07** | Cluster-35412.0 | Cluster-1785.4781 |
|  | n-acetylmuramoyl-l-alanine amidase-like | **-1.39** | 1.34 | Cluster-35821.0 | Cluster-34754.0 |
|  | acid trehalase-like protein 1 | -2.53 | **-0.17** | Cluster-35883.0 | Cluster-35094.1 |
|  | calcium-activated potassium channel subunit alpha-1 isoform x15 | 0.30 | **-4.30** | Cluster-36233.1 | Cluster-1785.5185 |
|  | armadillo repeat-containing protein 1-like | -4.79 | **-0.54** | Cluster-36654.0 | Cluster-34227.0 |
|  | calcium-binding mitochondrial carrier protein s -2 | **1.24** | -0.99 | Cluster-36691.10176 | Cluster-36070.0 |
|  | e3 ubiquitin-protein ligase rnf38-like | **-0.20** | 1.25 | Cluster-36691.12059 | Cluster-1785.2073 |
|  | serine threonine-protein kinase sgk1 isoform x2 | **1.81** | -0.23 | Cluster-36691.12300 | Cluster-19699.0 |
|  | vang-like protein 1 isoform x1 | **-0.62** | 1.65 | Cluster-36691.13553 | Cluster-1785.13804 |
|  | eosinophil peroxidase-like | **1.96** | -0.34 | Cluster-36691.13924 | Cluster-35546.0 |
|  | opsin- partial | **5.39** | -0.03 | Cluster-36691.15708 | Cluster-31586.0 |
|  | phosphatidylserine decarboxylase proenzyme isoform x3 | **-0.17** | -2.36 | Cluster-36691.1605 | Cluster-30331.2 |
|  | c-type lysozyme | -0.83 | 2.81 | Cluster-36691.16696 | Cluster-35516.1 |
|  | dimethylglycine mitochondrial | **-1.71** | 0.66 | Cluster-36691.2366 | Cluster-1785.13445 |
|  | amp deaminase 3-like isoform x1 | **-3.53** | 0.25 | Cluster-36691.4404 | Cluster-32372.0 |
|  | protein sprouty homolog 4 | **0.70** | -1.06 | Cluster-36691.5982 | Cluster-21080.0 |
|  | mhc class ia antigen | -2.37 | **0.65** | Cluster-36691.6532 | Cluster-1785.6660 |
|  | solute carrier family 25 member 33 | **0.41** | -1.50 | Cluster-36691.9006 | Cluster-1785.7150 |
|  | uridine-cytidine kinase 1 | -0.37 | 0.92 | Cluster-36691.9453 | Cluster-20927.0 |
|  | cyclin-dependent kinase 2-associated protein 1 isoform x1 | 0.48 | -1.88 | Cluster-7535.0 | Cluster-20778.0 |
|  | histone h2a | -1.32 | **0.08** | Cluster-7915.6 | Cluster-1785.5498 |
|  |  |  |  |  |  |
| *Low Temperature Challenge* | |  |  |  |  |
|  | guanine nucleotide-binding protein g subunit alpha isoform x1 | 1.22 | **-0.26** | Cluster-10173.0 | Cluster-30410.2 |
|  | ras-related protein rab-5a | 0.03 | **-0.98** | Cluster-10208.3 | Cluster-16032.1 |
|  | calcineurin b homologous protein 2-like | **-0.94** | **0.60** | Cluster-10385.1 | Cluster-35742.0 |
|  | metastasis-associated protein mta2 | -1.02 | **0.35** | Cluster-10764.0 | Cluster-23257.0 |
|  | testis-expressed sequence 2 protein | **-0.42** | **0.80** | Cluster-11213.0 | Cluster-28494.0 |
|  | transmembrane protein 53-like | 3.54 | **0.16** | Cluster-11398.0 | Cluster-1785.13892 |
|  | lim domain transcription factor lmo4-b-like | 0.76 | **-0.27** | Cluster-11820.0 | Cluster-21470.1 |
|  | ubiquitin carboxyl-terminal hydrolase 12-like | 2.84 | **1.51** | Cluster-11842.1 | Cluster-1785.10732 |
|  | immediate early response gene 2 | **0.44** | 2.44 | Cluster-12027.0 | Cluster-32585.0 |
|  | lysine-specific demethylase phf2 isoform x2 | **-0.96** | 0.69 | Cluster-1254.1 | Cluster-36533.0 |
|  | glutamate-rich wd repeat-containing protein 1 | -0.52 | 1.26 | Cluster-12729.0 | Cluster-34133.0 |
|  | leucyl-cystinyl aminopeptidase | 0.26 | **-1.08** | Cluster-13162.0 | Cluster-31732.0 |
|  | ferm domain-containing protein 4a isoform x1 | 1.47 | **-0.54** | Cluster-13414.0 | Cluster-20874.0 |
|  | endophilin-a2-like isoform x2 | **0.91** | -0.25 | Cluster-13467.0 | Cluster-14175.0 |
|  | at-rich interactive domain-containing protein 2 | **0.49** | -1.01 | Cluster-13496.0 | Cluster-17191.0 |
|  | transcriptional regulator myc-a-like | **0.70** | **3.06** | Cluster-13546.2 | Cluster-28076.0 |
|  | krueppel-like factor 8 isoform x1 | 0.78 | 2.10 | Cluster-13611.1 | Cluster-24212.0 |
|  | cysteine-rich secretory protein lccl domain-containing 2 | **-0.93** | **1.21** | Cluster-13630.0 | Cluster-22947.0 |
|  | ectoderm-neural cortex protein 1 | **-2.21** | -0.64 | Cluster-13752.0 | Cluster-1785.8094 |
|  | mki67 fha domain-interacting nucleolar phosphoprotein | **-0.27** | 1.16 | Cluster-13775.0 | Cluster-1785.9261 |
|  | krueppel-like factor 9 | -0.25 | 1.38 | Cluster-13863.0 | Cluster-16642.0 |
|  | ornithine mitochondrial | **3.59** | **0.77** | Cluster-13940.0 | Cluster-1785.13718 |
|  | transcriptional adapter 2-beta | -1.28 | **-0.17** | Cluster-13988.1 | Cluster-17730.1 |
|  | maltase- intestinal-like | **-3.04** | 0.28 | Cluster-14084.0 | Cluster-18400.0 |
|  | sodium-dependent neutral amino acid transporter b at2-like | **-1.53** | 0.15 | Cluster-14310.0 | Cluster-36230.0 |
|  | cyclin-g2-like isoform x1 | **1.18** | 0.27 | Cluster-14349.0 | Cluster-28385.0 |
|  | receptor-type tyrosine-protein phosphatase epsilon-like isoform x1 | -1.50 | **1.07** | Cluster-14431.0 | Cluster-23363.0 |
|  | serine palmitoyltransferase 2-like | **0.99** | -0.24 | Cluster-14498.0 | Cluster-28193.2 |
|  | myotubularin-related protein 6 | 0.59 | **-0.65** | Cluster-14501.0 | Cluster-23335.0 |
|  | lipid phosphate phosphohydrolase 3-like | **-0.59** | 1.08 | Cluster-14503.0 | Cluster-1785.6367 |
|  | protein cyr61-like | -0.82 | **2.09** | Cluster-14547.0 | Cluster-11381.0 |
|  | oxysterol-binding protein 3-like | **-0.21** | 0.87 | Cluster-14695.0 | Cluster-15714.0 |
|  | histone h4 transcription factor-like | **2.15** | 0.61 | Cluster-14892.1 | Cluster-35475.0 |
|  | glypican-5-like isoform x1 | -2.04 | **-0.33** | Cluster-14941.2 | Cluster-19413.0 |
|  | ring finger protein unkempt homolog | 0.17 | **-0.80** | Cluster-15462.0 | Cluster-1785.7034 |
|  | epoxide hydrolase 1 | 1.07 | **-0.05** | Cluster-156.3 | Cluster-25871.0 |
|  | xyloside xylosyltransferase 1 | **-1.10** | -2.72 | Cluster-15684.1 | Cluster-22129.0 |
|  | protein lifeguard 4 | **0.73** | -0.38 | Cluster-15753.0 | Cluster-1785.1941 |
|  | gsk3-beta interaction protein | 0.76 | **-0.75** | Cluster-15857.0 | Cluster-11326.0 |
|  | udp-xylose and udp-n-acetylglucosamine transporter | **1.44** | 0.06 | Cluster-16156.0 | Cluster-32599.1 |
|  | ekc keops complex subunit tprkb | -2.37 | **-0.03** | Cluster-16302.0 | Cluster-1785.14132 |
|  | secreted frizzled-related protein 5 | -3.17 | **0.23** | Cluster-16442.0 | Cluster-1785.9712 |
|  | succinate dehydrogenase | 3.10 | **1.69** | Cluster-16597.0 | Cluster-29875.0 |
|  | vesicle transport protein sft2a | 0.37 | -0.83 | Cluster-16613.2 | Cluster-35402.2 |
|  | parvalbumin beta-like | **-7.80** | **-0.44** | Cluster-17118.1 | Cluster-27211.2 |
|  | rho guanine nucleotide exchange factor 26 | **1.75** | -0.14 | Cluster-17261.1 | Cluster-1785.11482 |
|  | ras-related protein rab-3a | 1.96 | -0.90 | Cluster-17301.2 | Cluster-36434.0 |
|  | lanosterol synthase | **2.98** | -1.47 | Cluster-17331.2 | Cluster-1785.12841 |
|  | wd repeat-containing protein 82 | **-1.93** | **-0.42** | Cluster-17573.0 | Cluster-36060.0 |
|  | ankyrin repeat and socs box protein 6 | -1.24 | -0.05 | Cluster-17617.1 | Cluster-35159.0 |
|  | glutamine synthetase | **1.51** | **-0.02** | Cluster-17923.3 | Cluster-1785.2370 |
|  | retinoic acid receptor alpha-b-like | **-0.69** | 0.72 | Cluster-17991.1 | Cluster-23294.3 |
|  | estrogen receptor beta | **0.81** | -0.93 | Cluster-18017.2 | Cluster-1785.10259 |
|  | estrogen receptor beta-like isoform x2 | **1.30** | -1.06 | Cluster-18017.3 | Cluster-1785.2267 |
|  | mothers against decapentaplegic homolog 2 isoform x1 | -0.12 | 1.61 | Cluster-18253.0 | Cluster-29785.0 |
|  | kelch-like ech-associated protein 1 | 1.64 | **-0.92** | Cluster-18314.1 | Cluster-28987.0 |
|  | non-canonical poly rna polymerase papd7-like | 0.94 | **-0.44** | Cluster-18336.0 | Cluster-20557.0 |
|  | trinucleotide repeat-containing gene 18 protein isoform x1 | 1.08 | **4.07** | Cluster-18351.2 | Cluster-13748.0 |
|  | protein fam69a-like | -1.87 | **0.00** | Cluster-18360.0 | Cluster-26577.0 |
|  | protein phosphatase slingshot homolog 2-like isoform x1 | **0.58** | **1.78** | Cluster-18461.0 | Cluster-30172.0 |
|  | atp-dependent rna helicase ddx24 | -0.74 | **0.47** | Cluster-18482.1 | Cluster-1785.5244 |
|  | g-protein coupled receptor 161 | 2.00 | 0.16 | Cluster-18576.0 | Cluster-1785.9681 |
|  | cyclin-dependent kinase inhibitor 1-like isoform x2 | **2.04** | **0.87** | Cluster-18654.0 | Cluster-1785.5851 |
|  | alpha beta hydrolase domain-containing protein 17b | **0.39** | -0.64 | Cluster-18770.1 | Cluster-34576.0 |
|  | bromodomain-containing protein 1-like isoform x1 | 3.04 | 0.74 | Cluster-18851.0 | Cluster-21813.0 |
|  | atp-binding cassette sub-family g member 2 | **2.31** | -0.91 | Cluster-1893.3 | Cluster-31517.1 |
|  | protein phosphatase slingshot homolog 2-like isoform x2 | **-0.87** | 0.42 | Cluster-18952.0 | Cluster-8734.1 |
|  | g-protein-signaling modulator 2 isoform x1 | 0.28 | -1.09 | Cluster-18996.1 | Cluster-25742.0 |
|  | xk-related protein 2 | 0.85 | **-0.61** | Cluster-19082.0 | Cluster-29624.1 |
|  | mitochondrial ubiquitin ligase activator of nfkb 1-a-like | -0.24 | **-1.51** | Cluster-19185.0 | Cluster-26966.0 |
|  | rho gtpase-activating protein syde2 | **-0.95** | 0.16 | Cluster-19291.0 | Cluster-10032.0 |
|  | hyaluronidase-2-like | **-1.14** | 1.11 | Cluster-19343.0 | Cluster-35305.0 |
|  | cyclic amp-dependent transcription factor atf-4-like | -0.52 | **0.50** | Cluster-19382.0 | Cluster-18223.0 |
|  | zinc transporter 4 | 1.17 | -0.06 | Cluster-19438.3 | Cluster-1785.8764 |
|  | zinc-binding protein a33-like | **-0.67** | -1.66 | Cluster-19625.1 | Cluster-34993.0 |
|  | stonustoxin subunit alpha-like | 2.44 | -1.40 | Cluster-19632.0 | Cluster-28696.1 |
|  | long-chain fatty acid transport protein 4 | -2.39 | **0.78** | Cluster-19648.0 | Cluster-15603.0 |
|  | catenin delta-1-like isoform x3 | **0.00** | -1.39 | Cluster-19662.1 | Cluster-1785.6008 |
|  | 7-methylguanosine phosphate-specific 5 -nucleotidase | **2.63** | 0.63 | Cluster-19726.0 | Cluster-26833.1 |
|  | recombining binding protein suppressor of hairless isoform x1 | -1.11 | **2.56** | Cluster-19957.0 | Cluster-21204.0 |
|  | pleiotropic regulator 1 | -0.31 | 1.77 | Cluster-20079.1 | Cluster-1785.10672 |
|  | e3 ubiquitin-protein ligase rnf170-like | 1.38 | **-1.16** | Cluster-20124.1 | Cluster-33440.0 |
|  | 3-hydroxyisobutyrate mitochondrial-like | -0.57 | **-1.55** | Cluster-20186.0 | Cluster-13995.0 |
|  | apelin | 1.17 | -0.23 | Cluster-20211.0 | Cluster-18302.0 |
|  | phd finger protein 20 isoform x1 | 0.18 | **1.39** | Cluster-20266.0 | Cluster-19301.0 |
|  | transcriptional regulator myc-like | **-1.93** | 0.54 | Cluster-20279.0 | Cluster-22873.0 |
|  | enolase-phosphatase e1-like isoform x3 | -1.39 | **0.34** | Cluster-20406.0 | Cluster-1785.13261 |
|  | signal-induced proliferation-associated 1-like protein 1 | **0.46** | -1.15 | Cluster-20433.1 | Cluster-21695.0 |
|  | ubiquitin thioesterase zranb1 | -1.05 | **0.30** | Cluster-20447.1 | Cluster-1785.11066 |
|  | transcription factor ap-1-like | -0.03 | **2.17** | Cluster-20474.0 | Cluster-26786.0 |
|  | atrial natriuretic peptide receptor 3 | 1.34 | **-0.29** | Cluster-20499.0 | Cluster-12609.0 |
|  | frizzled-8 | **-2.32** | **-0.55** | Cluster-20508.0 | Cluster-30599.2 |
|  | cyclin-l1 isoform x2 | 0.43 | **1.70** | Cluster-20848.0 | Cluster-1785.574 |
|  | transforming growth factor-beta-induced protein ig-h3 | **-1.26** | 0.94 | Cluster-20853.0 | Cluster-16306.0 |
|  | phd finger protein 12 | **-0.64** | -1.64 | Cluster-20925.0 | Cluster-36631.0 |
|  | glia-derived nexin | **0.90** | 2.89 | Cluster-20934.0 | Cluster-33939.0 |
|  | monocyte to macrophage differentiation factor 2-like | -0.25 | -2.41 | Cluster-20941.0 | Cluster-23809.0 |
|  | cyclin-j-like protein | **0.23** | -1.43 | Cluster-20977.0 | Cluster-1785.3993 |
|  | group xiib secretory phospholipase a2-like protein isoform x2 | 1.07 | **-0.60** | Cluster-21003.1 | Cluster-1785.5199 |
|  | matrix metalloproteinase-15-like | **0.53** | **-0.98** | Cluster-21036.0 | Cluster-19908.0 |
|  | ran gtpase-activating protein 1-like | -0.29 | **1.23** | Cluster-21047.0 | Cluster-18756.0 |
|  | translocator protein | **0.96** | 0.02 | Cluster-21088.0 | Cluster-20619.0 |
|  | cyclin-dependent kinase inhibitor 1-like isoform x1 | **-0.52** | **1.15** | Cluster-21112.0 | Cluster-25110.2 |
|  | high affinity cationic amino acid transporter 1-like isoform x1 | 1.72 | **-0.52** | Cluster-21116.1 | Cluster-1785.1348 |
|  | histone -b | 1.58 | **-0.37** | Cluster-21123.0 | Cluster-26432.0 |
|  | rna pseudouridylate synthase domain-containing protein 4-like | 0.17 | **-1.11** | Cluster-21135.0 | Cluster-15587.3 |
|  | neuronal acetylcholine receptor subunit alpha-7 | -3.10 | **-0.74** | Cluster-21151.0 | Cluster-15438.0 |
|  | mitochondrial | **1.06** | -0.45 | Cluster-21218.1 | Cluster-8009.0 |
|  | arf-gap with ank repeat and ph domain-containing protein 3 isoform x1 | 0.93 | **-0.55** | Cluster-21225.1 | Cluster-19642.0 |
|  | transposon ty3-i gag-pol polyprotein | -2.95 | 0.35 | Cluster-2125.4 | Cluster-5076.1 |
|  | phosphatidylinositol 3-kinase regulatory subunit gamma-like isoform x2 | -2.00 | **-0.31** | Cluster-21255.0 | Cluster-20853.0 |
|  | egl nine homolog 1 | 0.80 | **-0.32** | Cluster-21263.0 | Cluster-31878.0 |
|  | solute carrier family 25 member 38-a-like | 0.30 | 2.94 | Cluster-21386.0 | Cluster-1785.5390 |
|  | chromobox protein homolog 3-like | **0.09** | 1.24 | Cluster-21422.0 | Cluster-10704.0 |
|  | protein-l-isoaspartate o-methyltransferase domain-containing protein 1 | **-1.00** | 0.78 | Cluster-21485.0 | Cluster-1785.3696 |
|  | rho guanine nucleotide exchange factor 17 | 0.80 | **-0.62** | Cluster-21520.0 | Cluster-1785.9343 |
|  | ap-3 complex subunit sigma-1 isoform x1 | **-0.26** | **1.11** | Cluster-21526.0 | Cluster-19119.0 |
|  | u11 u12 small nuclear ribonucleoprotein 35 kda protein | -1.32 | 1.17 | Cluster-21550.0 | Cluster-21628.0 |
|  | choline transporter-like protein 2 isoform x2 | **0.10** | -1.01 | Cluster-21576.0 | Cluster-35467.0 |
|  | ectonucleoside triphosphate diphosphohydrolase 1 isoform x1 | **-0.52** | 0.65 | Cluster-21653.0 | Cluster-1785.1670 |
|  | protein ndrg2 | **-0.36** | 0.99 | Cluster-21673.0 | Cluster-15266.0 |
|  | pseudouridylate synthase 7 homolog isoform x2 | 0.09 | **1.08** | Cluster-21714.0 | Cluster-27890.2 |
|  | ras-related protein rab-9b-like | **-0.30** | 0.94 | Cluster-21727.0 | Cluster-31377.0 |
|  | 5-aminolevulinate erythroid- mitochondrial | -0.80 | **0.58** | Cluster-21730.0 | Cluster-20041.1 |
|  | dynein light chain axonemal | **-1.01** | -2.71 | Cluster-21772.0 | Cluster-21920.0 |
|  | nicotinamide nicotinic acid mononucleotide adenylyltransferase 1 | 0.62 | -0.72 | Cluster-21807.0 | Cluster-32631.0 |
|  | large neutral amino acids transporter small subunit 2-like | **-0.29** | 2.56 | Cluster-21834.1 | Cluster-24554.0 |
|  | b-cell cll lymphoma 7 protein family member a isoform x1 | **4.57** | -1.30 | Cluster-21865.0 | Cluster-35658.0 |
|  | phospholipase d1-like | 0.13 | -0.92 | Cluster-21920.2 | Cluster-1785.12293 |
|  | probable atp-dependent rna helicase ddx10 | **0.24** | **1.65** | Cluster-21988.0 | Cluster-22541.0 |
|  | uncharacterized oxidoreductase -like | **-0.17** | -1.36 | Cluster-22155.0 | Cluster-35517.0 |
|  | microphthalmia-associated transcription factor isoform x2 | -0.02 | **-1.79** | Cluster-22209.1 | Cluster-23618.0 |
|  | syntaxin-3 isoform x3 | **-0.25** | **-1.64** | Cluster-22221.0 | Cluster-18608.0 |
|  | ankyrin repeat domain-containing protein 50 | **-1.41** | 0.04 | Cluster-22312.0 | Cluster-26562.0 |
|  | 39s ribosomal protein mitochondrial isoform x2 | 0.94 | **-1.53** | Cluster-22316.0 | Cluster-24574.1 |
|  | poly | -0.99 | 3.60 | Cluster-22335.0 | Cluster-12239.8 |
|  | proto-oncogene serine threonine-protein kinase mos | **-2.72** | -0.03 | Cluster-22461.0 | Cluster-22251.0 |
|  | transcriptional coactivator yap1 | **-0.03** | **1.18** | Cluster-22465.0 | Cluster-29526.0 |
|  | arf-gap with rho-gap ank repeat and ph domain-containing protein 2 | **-1.15** | 0.38 | Cluster-22505.0 | Cluster-35420.0 |
|  | at-rich interactive domain-containing protein 4b isoform x1 | 0.95 | **-0.51** | Cluster-22508.0 | Cluster-13735.1 |
|  | enhancer of mrna-decapping protein 3 | **-1.71** | -3.02 | Cluster-22531.0 | Cluster-23776.0 |
|  | map kinase-interacting serine threonine-protein kinase 2 | 2.39 | **0.61** | Cluster-22591.0 | Cluster-1785.13803 |
|  | aprataxin isoform x1 | **-1.74** | -0.22 | Cluster-22688.0 | Cluster-21931.0 |
|  | transmembrane protein 222-like | 0.32 | **-0.82** | Cluster-22703.0 | Cluster-20633.0 |
|  | peroxisome proliferator-activated receptor alpha-like | **1.85** | -0.61 | Cluster-22754.0 | Cluster-1785.3119 |
|  | transmembrane protein 53 | **-0.19** | -1.88 | Cluster-22795.0 | Cluster-8255.0 |
|  | forkhead box protein k2-like | **-1.78** | 0.44 | Cluster-228.5 | Cluster-14483.0 |
|  | solute carrier family 12 member 7-like isoform x1 | 0.44 | **-1.03** | Cluster-22815.3 | Cluster-1785.4467 |
|  | peroxisomal leader peptide-processing protease | **-1.81** | 0.33 | Cluster-22994.1 | Cluster-14443.0 |
|  | transmembrane protein 60-like | **-0.07** | 1.18 | Cluster-23018.0 | Cluster-26003.0 |
|  | ets domain-containing protein elk-3-like | **0.72** | **-0.20** | Cluster-23055.0 | Cluster-29239.0 |
|  | e3 ubiquitin-protein ligase nedd4 isoform x1 | **0.79** | 2.45 | Cluster-23071.0 | Cluster-15577.1 |
|  | sperm-specific antigen 2 isoform x1 | **0.55** | 1.66 | Cluster-23120.0 | Cluster-1785.4595 |
|  | g-protein coupled receptor 39 | 0.74 | **-0.71** | Cluster-23126.1 | Cluster-9923.0 |
|  | alpha-actinin-1 isoform x3 | **0.56** | -0.60 | Cluster-23195.10 | Cluster-1785.7641 |
|  | 28s ribosomal protein mitochondrial | **0.86** | -0.11 | Cluster-23224.0 | Cluster-25173.0 |
|  | iron-sulfur cluster assembly enzyme mitochondrial | **-1.83** | **-0.30** | Cluster-23247.0 | Cluster-10031.0 |
|  | afg3-like protein 1 isoform x1 | **0.96** | -0.35 | Cluster-23577.0 | Cluster-7121.0 |
|  | pdz and lim domain protein 1 | **2.24** | 0.22 | Cluster-23600.0 | Cluster-33817.0 |
|  | reticulon-4 receptor | **-1.03** | -3.05 | Cluster-23653.0 | Cluster-11119.0 |
|  | homeobox protein meis1 | **-1.57** | -0.18 | Cluster-23756.0 | Cluster-34284.0 |
|  | lysophosphatidic acid receptor 2 | 1.52 | -1.49 | Cluster-23768.1 | Cluster-1785.718 |
|  | protein phosphatase 1 regulatory subunit 3c | 1.00 | -1.09 | Cluster-23938.0 | Cluster-1785.13116 |
|  | solute carrier family 12 member 4 | 1.69 | **0.30** | Cluster-23971.1 | Cluster-5117.0 |
|  | short-chain dehydrogenase reductase 3 | -1.08 | 0.76 | Cluster-24045.0 | Cluster-25586.0 |
|  | endophilin-a2-like isoform x2 | -1.29 | -0.28 | Cluster-24049.0 | Cluster-35516.2 |
|  | tumor necrosis factor ligand superfamily member 11 | -2.68 | **-1.26** | Cluster-24105.2 | Cluster-34534.0 |
|  | proprotein convertase subtilisin kexin type 7 | 0.65 | **-0.59** | Cluster-24126.0 | Cluster-1785.9615 |
|  | dna-directed rna polymerase i subunit rpa12 | -0.28 | **1.37** | Cluster-24146.0 | Cluster-1785.5783 |
|  | lecithin retinol acyltransferase-like | -2.09 | **0.01** | Cluster-24164.1 | Cluster-34012.3 |
|  | ribokinase | **-1.16** | 0.51 | Cluster-24218.2 | Cluster-11908.0 |
|  | coiled-coil domain-containing protein 94 | 0.26 | **-2.90** | Cluster-24229.0 | Cluster-27737.0 |
|  | transcription factor jun-b-like | 1.12 | **3.09** | Cluster-24271.0 | Cluster-19790.0 |
|  | disintegrin and metalloproteinase domain-containing protein 10-like | **0.62** | -0.84 | Cluster-24320.0 | Cluster-1785.13486 |
|  | hepatic leukemia factor-like | **1.42** | **-0.22** | Cluster-24407.0 | Cluster-23406.0 |
|  | disks large-associated protein 1 isoform x1 | **-2.25** | **0.61** | Cluster-24527.0 | Cluster-24282.0 |
|  | cell death activator cide-3-like | **0.86** | **2.23** | Cluster-24550.2 | Cluster-24615.2 |
|  | 2-aminoethanethiol dioxygenase-like | **-0.32** | **-2.16** | Cluster-24558.1 | Cluster-31495.0 |
|  | sphingosine kinase 2-like | **0.90** | 2.70 | Cluster-24649.1 | Cluster-1785.46 |
|  | receptor-type tyrosine-protein phosphatase mu-like | -0.35 | **2.14** | Cluster-24673.0 | Cluster-1785.8785 |
|  | growth arrest and dna damage-inducible proteins-interacting protein 1 | -1.31 | 0.82 | Cluster-24910.0 | Cluster-1785.13516 |
|  | cell division cycle-associated 7-like protein | 1.40 | -0.12 | Cluster-25048.0 | Cluster-27361.1 |
|  | heterogeneous nuclear ribonucleoprotein c-like isoform x1 | **-1.02** | -2.60 | Cluster-2506.1 | Cluster-1785.11350 |
|  | eukaryotic translation initiation factor 4 gamma 3 isoform x3 | -1.28 | 0.08 | Cluster-25193.0 | Cluster-33144.0 |
|  | PREDICTED: uncharacterized protein K02A2.6-like | -1.37 | 0.28 | Cluster-25274.3 | Cluster-1785.886 |
|  | ubiquitin-fold modifier-conjugating enzyme 1 | **-0.47** | 0.87 | Cluster-25275.0 | Cluster-35260.0 |
|  | adp-ribosylation factor-like protein 5b | -0.02 | -1.24 | Cluster-25287.0 | Cluster-1785.10917 |
|  | pre-mrna-splicing factor spf27 | 0.10 | **1.16** | Cluster-25395.0 | Cluster-2931.1 |
|  | dna-directed rna polymerase iii subunit rpc5 | -0.30 | **0.85** | Cluster-25446.0 | Cluster-1785.5038 |
|  | cationic amino acid transporter 2 isoform x1 | **1.74** | **-0.30** | Cluster-25449.4 | Cluster-1785.4912 |
|  | rap guanine nucleotide exchange factor 5-like | 3.65 | **1.56** | Cluster-25550.1 | Cluster-33340.1 |
|  | beta-sarcoglycan | **0.59** | -0.35 | Cluster-25594.0 | Cluster-19111.0 |
|  | transcription initiation factor tfiid subunit 5 | **-1.49** | **-2.93** | Cluster-25642.1 | Cluster-2529.1 |
|  | phosphatidylinositol n-acetylglucosaminyltransferase subunit a | 0.08 | **-1.43** | Cluster-25697.1 | Cluster-27289.0 |
|  | rho gtpase-activating protein 6-like isoform x2 | -1.73 | **-0.09** | Cluster-25746.0 | Cluster-29632.0 |
|  | dehydrogenase reductase sdr family member 11 | 2.60 | **0.77** | Cluster-25764.0 | Cluster-30921.0 |
|  | vesicle-associated membrane protein 7 | **0.89** | **2.46** | Cluster-25789.0 | Cluster-24657.0 |
|  | g1 s-specific cyclin-e1 | -0.26 | **1.56** | Cluster-25840.1 | Cluster-35221.0 |
|  | pleckstrin homology-like domain family a member 2 | **2.91** | **0.16** | Cluster-25903.0 | Cluster-35996.0 |
|  | septin-10-like isoform x2 | **0.63** | **-0.33** | Cluster-25924.1 | Cluster-1785.5410 |
|  | probable dimethyladenosine transferase | 0.30 | **1.38** | Cluster-25944.0 | Cluster-19482.1 |
|  | transgelin-like | 0.03 | **1.04** | Cluster-25986.0 | Cluster-36302.1 |
|  | dnaj homolog subfamily a member 4 | -0.59 | **1.98** | Cluster-26023.0 | Cluster-23282.0 |
|  | h(+) cl(-) exchange transporter 5 isoform x1 | 2.16 | **-0.10** | Cluster-26025.0 | Cluster-27823.0 |
|  | vegetative incompatibility protein het-e-1-like | -0.98 | **0.34** | Cluster-26039.2 | Cluster-1785.11592 |
|  | protein aatf | **-0.59** | 1.22 | Cluster-26175.0 | Cluster-1785.1590 |
|  | eukaryotic translation initiation factor 4e-like | -0.24 | **2.82** | Cluster-26191.1 | Cluster-12129.1 |
|  | gamma-glutamylaminecyclotransferase b-like | 0.86 | **-0.52** | Cluster-26214.0 | Cluster-18363.0 |
|  | water dikinase 1 | **1.19** | -0.40 | Cluster-26293.0 | Cluster-33761.1 |
|  | vitamin d3 receptor a | **2.54** | -0.86 | Cluster-26308.1 | Cluster-1785.8360 |
|  | sodium potassium-transporting atpase subunit beta-1 | 1.93 | 0.21 | Cluster-26346.1 | Cluster-25067.0 |
|  | e3 ubiquitin-protein ligase rad18 | **-1.02** | 0.76 | Cluster-26465.0 | Cluster-9478.0 |
|  | fatty acid 2-hydroxylase | **-0.04** | -1.30 | Cluster-26470.0 | Cluster-25313.0 |
|  | aminoacylase-1 | -0.66 | **0.31** | Cluster-26543.1 | Cluster-26549.0 |
|  | meckelin isoform x2 | -1.66 | **-0.11** | Cluster-26571.0 | Cluster-19092.0 |
|  | heparan sulfate glucosamine 3-o-sulfotransferase 6-like | 0.77 | **-1.28** | Cluster-26715.0 | Cluster-23590.4 |
|  | triosephosphate isomerase | **1.49** | -0.32 | Cluster-26751.0 | Cluster-25097.0 |
|  | suppressor of cytokine signaling 2 | -1.84 | **0.70** | Cluster-26807.1 | Cluster-1785.13520 |
|  | venom phosphodiesterase 1-like | **0.55** | **-1.24** | Cluster-26898.0 | Cluster-1785.5434 |
|  | protein tex261 | **-0.41** | **-1.75** | Cluster-26957.0 | Cluster-14893.0 |
|  | aspartate beta-hydroxylase domain-containing protein 2 | **-0.01** | -0.98 | Cluster-26994.0 | Cluster-25476.0 |
|  | u3 small nucleolar rna-associated protein 14 homolog a-like | -0.69 | **0.78** | Cluster-27032.0 | Cluster-30861.0 |
|  | methylmalonyl- mitochondrial | 0.16 | **-1.06** | Cluster-27042.0 | Cluster-1785.11094 |
|  | tom1-like protein 2 isoform x1 | **0.36** | -0.76 | Cluster-27094.0 | Cluster-1785.9309 |
|  | long-chain-fatty-acid-- ligase acsbg2-like | 1.04 | -1.00 | Cluster-27220.3 | Cluster-34903.1 |
|  | membrane protein fam174b-like | -0.75 | **0.38** | Cluster-27239.1 | Cluster-17846.0 |
|  | acetyl-coenzyme a cytoplasmic isoform x2 | **-0.29** | -1.47 | Cluster-27245.2 | Cluster-1785.10843 |
|  | tubulin beta chain-like | -1.36 | -0.02 | Cluster-27271.1 | Cluster-6091.2 |
|  | ubiquitin-like protein 3 | **1.33** | **0.15** | Cluster-27391.0 | Cluster-33496.0 |
|  | 2-hydroxyacylsphingosine 1-beta-galactosyltransferase | 0.44 | **-1.46** | Cluster-27399.0 | Cluster-32904.1 |
|  | rna-binding protein mex3b-like | **0.30** | 1.63 | Cluster-27412.1 | Cluster-21370.0 |
|  | class e basic helix-loop-helix protein 40-like | **1.71** | 0.64 | Cluster-27449.0 | Cluster-21705.0 |
|  | forkhead box protein j3-like | **0.91** | -0.75 | Cluster-27534.0 | Cluster-1785.13256 |
|  | selenocysteine-specific elongation factor | **-0.66** | -1.91 | Cluster-27573.0 | Cluster-17061.0 |
|  | ras-related protein rab-17-like | -0.76 | **1.04** | Cluster-27574.0 | Cluster-12848.0 |
|  | n-acetylglucosamine-1-phosphotransferase subunits alpha beta | -0.40 | **0.58** | Cluster-27643.0 | Cluster-22489.0 |
|  | endoplasmic reticulum aminopeptidase 1 | 0.61 | **-0.45** | Cluster-27789.0 | Cluster-22492.0 |
|  | plasma alpha-l-fucosidase | **-0.22** | 1.16 | Cluster-27908.0 | Cluster-19552.0 |
|  | tp53-regulating kinase | -1.31 | **-0.10** | Cluster-27912.0 | Cluster-25840.0 |
|  | protein cyr61 | **-0.26** | **3.26** | Cluster-27915.0 | Cluster-26134.0 |
|  | lysozyme g-like | -0.07 | **1.59** | Cluster-27976.0 | Cluster-29549.0 |
|  | aspartate cytoplasmic-like | **1.74** | **0.20** | Cluster-28059.0 | Cluster-33193.0 |
|  | muscarinic acetylcholine receptor m5-like | -3.64 | -0.77 | Cluster-28141.0 | Cluster-26038.0 |
|  | coronin-7-like isoform x1 | **-0.35** | 1.07 | Cluster-28156.0 | Cluster-5744.0 |
|  | endothelial pas domain-containing protein 1 | 1.44 | **-0.35** | Cluster-28187.0 | Cluster-1944.0 |
|  | syndecan-4-like isoform x1 | 0.68 | **2.52** | Cluster-28454.0 | Cluster-19806.0 |
|  | elmo domain-containing protein 2 | 1.00 | **-0.17** | Cluster-28456.1 | Cluster-19780.0 |
|  | suppressor of cytokine signaling 3 | 1.61 | 3.83 | Cluster-28495.0 | Cluster-23254.0 |
|  | eukaryotic translation initiation factor 5 | -0.54 | 0.73 | Cluster-28549.0 | Cluster-35353.0 |
|  | dna damage-inducible transcript 3 protein | **1.07** | 3.63 | Cluster-28552.1 | Cluster-26108.0 |
|  | e3 ubiquitin-protein ligase cbl-b | **1.25** | **-0.73** | Cluster-28565.2 | Cluster-21960.2 |
|  | olfactory receptor 13c8-like | -1.93 | **0.64** | Cluster-28572.0 | Cluster-34158.1 |
|  | oxysterols receptor lxr-alpha | **0.61** | -0.99 | Cluster-28577.0 | Cluster-28354.0 |
|  | at-rich interactive domain-containing protein 5b-like | **-1.39** | 2.12 | Cluster-28625.0 | Cluster-17279.0 |
|  | adp atp translocase 2 | 1.24 | -1.57 | Cluster-28654.1 | Cluster-25582.2 |
|  | cytochrome p450 1a | 0.75 | **-1.30** | Cluster-28668.0 | Cluster-22612.0 |
|  | bag family molecular chaperone regulator 4 | **0.41** | -0.94 | Cluster-28696.0 | Cluster-20039.0 |
|  | 60s ribosomal protein l7-like 1 | -0.21 | 0.92 | Cluster-28711.0 | Cluster-28737.0 |
|  | serine threonine-protein kinase kist | **-0.35** | -1.76 | Cluster-28766.1 | Cluster-33389.1 |
|  | erythrocyte band 7 integral membrane | 2.56 | -0.13 | Cluster-28795.0 | Cluster-15918.0 |
|  | pseudouridylate synthase 7 homolog-like protein | **-0.49** | **1.69** | Cluster-28855.0 | Cluster-1695.0 |
|  | wd repeat-containing protein 55 | -0.82 | **1.14** | Cluster-28883.0 | Cluster-1785.6005 |
|  | tropomyosin alpha-4 chain isoform x3 | 0.88 | **-0.10** | Cluster-29023.0 | Cluster-4004.4 |
|  | cytosolic 10-formyltetrahydrofolate dehydrogenase-like | **2.02** | -0.59 | Cluster-29055.1 | Cluster-18746.4 |
|  | xk-related protein 8-like | -0.67 | **1.28** | Cluster-29193.1 | Cluster-30373.2 |
|  | cmp-n-acetylneuraminate-beta-galactosamide-alpha- -sialyltransferase 1-like | 0.69 | **-1.38** | Cluster-29322.0 | Cluster-1785.11359 |
|  | mitochondrial basic amino acids transporter-like | **2.44** | 0.80 | Cluster-29447.0 | Cluster-1785.803 |
|  | chondroitin sulfate n-acetylgalactosaminyltransferase 2 | **-1.05** | **-2.42** | Cluster-29452.0 | Cluster-30701.1 |
|  | probable phospholipid-transporting atpase ih isoform x1 | -1.36 | 0.47 | Cluster-29474.0 | Cluster-32017.0 |
|  | exosome complex component rrp41 | **-0.53** | **-1.59** | Cluster-29488.0 | Cluster-24768.0 |
|  | plexin-b1-like isoform x1 | -1.05 | 0.32 | Cluster-29605.1 | Cluster-3448.1 |
|  | bile acid- :amino acid n-acyltransferase-like isoform x2 | **-1.53** | 2.42 | Cluster-29636.0 | Cluster-1785.4530 |
|  | ligand of numb protein x 2-like | **0.18** | -0.97 | Cluster-29704.2 | Cluster-1785.8535 |
|  | serine threonine-protein kinase sbk2-like | **0.33** | **-2.54** | Cluster-29746.0 | Cluster-36095.0 |
|  | phosphoglycerate mutase 1-like | **0.94** | **-0.54** | Cluster-29846.0 | Cluster-23100.0 |
|  | hand2 protein | 0.37 | -1.14 | Cluster-29954.0 | Cluster-22667.0 |
|  | serine threonine-protein kinase 35-like | 1.34 | **-0.04** | Cluster-29993.0 | Cluster-16280.2 |
|  | aspartate cytoplasmic | **1.82** | -0.04 | Cluster-30008.2 | Cluster-1785.8552 |
|  | yrdc domain-containing mitochondrial | **-1.40** | 1.17 | Cluster-30123.0 | Cluster-6284.0 |
|  | p53-induced death domain-containing protein 1 | **0.96** | -0.30 | Cluster-30131.0 | Cluster-30556.0 |
|  | sestrin-3-like isoform x1 | **0.67** | -0.47 | Cluster-30248.1 | Cluster-1785.6884 |
|  | low quality protein: nodal modulator 1-like | **1.82** | **0.31** | Cluster-30356.1 | Cluster-19584.0 |
|  | disintegrin and metalloproteinase domain-containing protein 9-like | **-0.52** | **1.39** | Cluster-30544.1 | Cluster-27511.0 |
|  | catechol o-methyltransferase domain-containing protein 1 isoform x1 | 1.67 | **-0.35** | Cluster-30545.0 | Cluster-1785.11596 |
|  | msx2-interacting protein | -0.30 | **-1.82** | Cluster-30566.0 | Cluster-34514.0 |
|  | uncharacterized family 31 glucosidase kiaa1161-like | **1.09** | -0.78 | Cluster-30578.0 | Cluster-26514.0 |
|  | cytochrome b-245 light chain | 0.01 | **1.58** | Cluster-30693.1 | Cluster-25499.0 |
|  | alpha- -mannosyl-glycoprotein 4-beta-n-acetylglucosaminyltransferase a | 0.71 | **-0.41** | Cluster-30802.1 | Cluster-33369.0 |
|  | paraspeckle component 1 isoform x3 | 0.67 | -1.03 | Cluster-30814.0 | Cluster-16296.0 |
|  | myosin heavy fast skeletal muscle-like | **-6.69** | 0.56 | Cluster-3098.15 | Cluster-17611.2 |
|  | zinc finger protein pegasus-like isoform x2 | **0.45** | -0.59 | Cluster-30999.0 | Cluster-18887.0 |
|  | glycogen debranching enzyme isoform x1 | 1.74 | **-0.14** | Cluster-31115.0 | Cluster-1785.12334 |
|  | trna-dihydrouridine synthase | **-0.72** | 0.18 | Cluster-31171.0 | Cluster-19645.0 |
|  | anthrax toxin receptor 1-like | 2.19 | **-0.32** | Cluster-31176.1 | Cluster-12493.2 |
|  | leucine-rich repeat transmembrane protein flrt3 | **-0.50** | -3.39 | Cluster-31191.0 | Cluster-19439.0 |
|  | acetoacetyl- synthetase | 2.94 | **0.32** | Cluster-31401.0 | Cluster-30907.0 |
|  | protein transport protein sec61 subunit alpha-like 1 | -1.51 | **0.18** | Cluster-31673.1 | Cluster-31045.2 |
|  | protein l-myc | 2.71 | **1.29** | Cluster-31681.0 | Cluster-23196.0 |
|  | nuclear receptor ror-alpha isoform x1 | 3.46 | **1.39** | Cluster-31730.0 | Cluster-31939.0 |
|  | erythropoietin receptor-like | **-0.62** | 1.11 | Cluster-31747.0 | Cluster-1785.2748 |
|  | utp--glucose-1-phosphate uridylyltransferase-like isoform x1 | **1.42** | -0.43 | Cluster-31877.0 | Cluster-1785.2355 |
|  | star-related lipid transfer protein mitochondrial | 0.44 | **-0.40** | Cluster-31918.0 | Cluster-25362.0 |
|  | tir domain-containing adapter molecule 1-like | -1.47 | **0.14** | Cluster-31975.0 | Cluster-12120.0 |
|  | nucleoporin p54 isoform x1 | -0.69 | **0.31** | Cluster-31991.0 | Cluster-1785.1371 |
|  | 39s ribosomal protein mitochondrial | 0.16 | **1.20** | Cluster-31992.0 | Cluster-18597.0 |
|  | proto-oncogene c-fos-like | -1.42 | 3.39 | Cluster-32096.0 | Cluster-24797.0 |
|  | glutaryl- mitochondrial-like | 2.54 | **-1.38** | Cluster-32121.0 | Cluster-24073.0 |
|  | hmg box-containing protein 1 | 0.88 | **-0.14** | Cluster-32273.0 | Cluster-20367.0 |
|  | e3 ubiquitin-protein ligase rnf8 isoform x2 | **0.41** | -2.34 | Cluster-32381.1 | Cluster-12737.1 |
|  | zdhhc-type palmitoyltransferase 6 isoform x1 | -1.72 | **0.27** | Cluster-32422.0 | Cluster-15442.0 |
|  | lix1-like protein | **1.73** | **0.28** | Cluster-32479.0 | Cluster-27534.1 |
|  | vesicle-associated membrane protein 8-like isoform x1 | -0.70 | **0.25** | Cluster-32679.3 | Cluster-1785.3813 |
|  | ceramide synthase 1 | **0.58** | -1.53 | Cluster-32699.1 | Cluster-19443.0 |
|  | fibroblast growth factor 1 isoform x2 | **0.42** | -0.75 | Cluster-32724.0 | Cluster-26047.0 |
|  | lysophospholipid acyltransferase 5 isoform x1 | -0.05 | **-1.35** | Cluster-32750.0 | Cluster-25219.0 |
|  | s-adenosyl-l-methionine-dependent trna 4-demethylwyosine synthase | **-0.87** | -2.11 | Cluster-32769.0 | Cluster-33867.0 |
|  | protein phosphatase 1 regulatory subunit 3b | -3.76 | **-0.64** | Cluster-32770.0 | Cluster-1785.13903 |
|  | male-specific lethal 1 homolog isoform x1 | **-0.31** | -1.30 | Cluster-32828.1 | Cluster-30914.1 |
|  | guanine nucleotide exchange factor dbs isoform x2 | -0.70 | 0.73 | Cluster-32880.0 | Cluster-30115.0 |
|  | fos-related antigen 2 | 0.95 | 3.59 | Cluster-32953.0 | Cluster-18130.0 |
|  | ring finger and chy zinc finger domain-containing protein1 | 0.41 | **-1.17** | Cluster-32958.0 | Cluster-1785.12076 |
|  | ubiquitin-protein ligase e3a | 1.31 | 2.77 | Cluster-33080.1 | Cluster-33941.1 |
|  | plasmalemma vesicle-associated protein | **-0.78** | 0.75 | Cluster-33262.1 | Cluster-1785.7302 |
|  | e3 ubiquitin-protein ligase pellino homolog 2 | **2.33** | 0.93 | Cluster-33295.0 | Cluster-12504.1 |
|  | zinc finger and btb domain-containing protein 21 | **0.16** | 1.45 | Cluster-33310.0 | Cluster-33592.1 |
|  | diphthamide biosynthesis protein 2 | -0.31 | **1.15** | Cluster-33336.0 | Cluster-30745.0 |
|  | pol polyprotein | **-3.62** | 0.83 | Cluster-3349.0 | Cluster-1785.12284 |
|  | cyclic amp-dependent transcription factor atf-3 | 2.50 | **5.94** | Cluster-33505.0 | Cluster-26463.0 |
|  | hepatocyte nuclear factor 3-beta-like | **0.41** | -0.67 | Cluster-33549.0 | Cluster-25507.0 |
|  | fidgetin-like | -1.31 | **0.44** | Cluster-33658.0 | Cluster-21898.0 |
|  | sulfhydryl oxidase 1 | **0.14** | **-1.51** | Cluster-33695.0 | Cluster-34883.0 |
|  | flavin-containing monooxygenase fmo gs-ox5-like | **0.15** | -1.37 | Cluster-33725.1 | Cluster-1785.3256 |
|  | large neutral amino acids transporter small subunit 3-like | 2.12 | **-0.16** | Cluster-33888.0 | Cluster-31372.1 |
|  | transforming growth factor-beta receptor-associated protein 1 | 0.37 | -0.89 | Cluster-33942.1 | Cluster-23929.1 |
|  | probable atp-dependent rna helicase ddx5 isoform x1 | **0.14** | **1.55** | Cluster-33955.3 | Cluster-28476.1 |
|  | solute carrier family 12 member 6 isoform x1 | **1.00** | -0.29 | Cluster-3397.12 | Cluster-1785.7059 |
|  | nad-dependent protein deacetylase sirtuin-1 | **-0.15** | 0.94 | Cluster-34021.0 | Cluster-27701.0 |
|  | rho-related btb domain-containing protein 2-like isoform x2 | **0.47** | -1.55 | Cluster-34065.1 | Cluster-21190.1 |
|  | sulfate transporter | 0.68 | **-1.67** | Cluster-34107.0 | Cluster-1785.2624 |
|  | transcription factor ap-4 isoform x1 | **-0.75** | 0.66 | Cluster-34152.1 | Cluster-29297.0 |
|  | swi snf-related matrix-associated actin-dependent regulator of chromatin subfamily a member 5 | **0.73** | 2.15 | Cluster-34219.0 | Cluster-30630.0 |
|  | ankyrin repeat and socs box protein 13-like | 0.36 | -1.03 | Cluster-34279.0 | Cluster-1785.13215 |
|  | methyltransferase ddb_g0268948 | -1.08 | 1.01 | Cluster-34309.2 | Cluster-31200.0 |
|  | growth factor receptor-bound protein 14 isoform x1 | 1.53 | -0.58 | Cluster-34327.0 | Cluster-24456.0 |
|  | eukaryotic initiation factor 4a-ii isoform x3 | -1.07 | **-0.13** | Cluster-34335.0 | Cluster-26881.1 |
|  | inhibin alpha chain | -4.39 | **0.19** | Cluster-34370.0 | Cluster-1785.2487 |
|  | alpha-catulin isoform x1 | **-0.84** | 0.09 | Cluster-34415.0 | Cluster-24911.0 |
|  | atp-dependent rna helicase dhx33 | 0.48 | **1.66** | Cluster-34422.0 | Cluster-36520.0 |
|  | phospholipid-transporting atpase ib isoform x3 | 0.48 | **-0.77** | Cluster-34447.1 | Cluster-25875.0 |
|  | plakophilin-1-like | -1.60 | **-0.29** | Cluster-34553.0 | Cluster-25077.1 |
|  | vinexin-like isoform x1 | **0.33** | -1.31 | Cluster-34586.0 | Cluster-30547.0 |
|  | platelet-derived growth factor receptor beta | **0.54** | -0.58 | Cluster-34616.0 | Cluster-1785.3108 |
|  | myotubularin-related protein 14 isoform x1 | **0.67** | **-0.42** | Cluster-34649.0 | Cluster-23359.1 |
|  | cdk5 and abl1 enzyme substrate 1 isoform x1 | **-0.32** | -1.64 | Cluster-34759.0 | Cluster-31184.0 |
|  | potassium voltage-gated channel subfamily kqt member 1-like | 1.77 | -1.05 | Cluster-34896.1 | Cluster-17945.0 |
|  | troponin slow skeletal muscle-like isoform x1 | -2.97 | **0.77** | Cluster-34906.1 | Cluster-1785.1948 |
|  | btb poz domain-containing adapter for cul3-mediated degradation protein 1 | **0.37** | **-0.71** | Cluster-34936.0 | Cluster-22576.0 |
|  | mpv17-like protein 2 | 0.89 | **-0.17** | Cluster-34980.0 | Cluster-14994.0 |
|  | inhibin beta b chain-like | **-4.21** | **3.48** | Cluster-34994.0 | Cluster-23161.0 |
|  | syntaxin-6 | **0.31** | -1.13 | Cluster-35045.0 | Cluster-1785.2674 |
|  | probable crossover junction endonuclease eme2 isoform x1 | **-2.19** | -0.76 | Cluster-35076.0 | Cluster-22215.0 |
|  | mediator of rna polymerase ii transcription subunit 17 | -0.93 | 0.03 | Cluster-35100.2 | Cluster-1785.13812 |
|  | transcription factor e2f1 | **0.82** | 2.52 | Cluster-35209.0 | Cluster-35522.0 |
|  | protein-methionine sulfoxide oxidase mical2 isoform x5 | -1.48 | **-2.86** | Cluster-35301.0 | Cluster-1785.444 |
|  | tyrosine-protein kinase fes fps | -1.52 | 1.62 | Cluster-35452.0 | Cluster-35872.1 |
|  | e3 ubiquitin-protein ligase trim39-like | -0.95 | **-4.10** | Cluster-35477.0 | Cluster-11471.0 |
|  | myocardial zonula adherens isoform x1 | **-0.30** | 1.05 | Cluster-35638.0 | Cluster-33540.0 |
|  | ras-related protein rab-9a-like | 1.26 | **-0.33** | Cluster-35693.0 | Cluster-36503.3 |
|  | sorting nexin-24 | **-1.14** | 0.08 | Cluster-35703.1 | Cluster-1785.1322 |
|  | heterogeneous nuclear ribonucleoprotein q isoform x1 | 0.00 | **1.17** | Cluster-35791.1 | Cluster-11321.1 |
|  | acid trehalase-like protein 1 | **0.70** | -0.38 | Cluster-35883.0 | Cluster-35094.1 |
|  | bmp and activin membrane-bound inhibitor homolog | **0.46** | -1.84 | Cluster-35900.3 | Cluster-34871.0 |
|  | endonuclease iii-like protein 1 | -1.91 | **0.07** | Cluster-35922.0 | Cluster-24289.1 |
|  | otu domain-containing protein 4 | **0.48** | **-0.91** | Cluster-35947.0 | Cluster-35175.0 |
|  | mbt domain-containing protein 1 isoform x1 | -0.70 | **-2.18** | Cluster-35961.0 | Cluster-24281.0 |
|  | protein enabled homolog isoform x2 | -0.58 | **1.13** | Cluster-35978.1 | Cluster-21661.0 |
|  | xk-related protein 9 | 3.93 | -1.13 | Cluster-36044.0 | Cluster-18948.0 |
|  | period circadian protein homolog 1 | 3.88 | 1.30 | Cluster-36073.0 | Cluster-17766.0 |
|  | little elongation complex subunit 2 | **-0.26** | 1.38 | Cluster-36093.0 | Cluster-35194.0 |
|  | transmembrane protein 161b | -0.35 | **-1.61** | Cluster-36142.0 | Cluster-19074.0 |
|  | ctp synthase 1-like | **0.69** | -0.56 | Cluster-36241.0 | Cluster-30221.0 |
|  | transmembrane and coiled-coil domains protein 1-like isoform x1 | 0.02 | -1.01 | Cluster-36344.0 | Cluster-14481.0 |
|  | rab11 family-interacting protein 3 isoform x2 | **0.52** | -1.41 | Cluster-36392.0 | Cluster-22622.0 |
|  | integrin-linked kinase-associated serine threonine phosphatase 2c | **-1.31** | **0.46** | Cluster-36407.3 | Cluster-1785.4490 |
|  | lactation elevated protein 1 | -2.14 | **-0.67** | Cluster-36437.1 | Cluster-25395.0 |
|  | alkaline ceramidase 2 | -0.40 | **1.84** | Cluster-36495.0 | Cluster-14106.0 |
|  | polycystic kidney disease protein 1-like 2 | **0.80** | **-1.46** | Cluster-36509.0 | Cluster-27256.0 |
|  | glycine dehydrogenase mitochondrial | 1.58 | **-0.64** | Cluster-36522.1 | Cluster-1785.10977 |
|  | adenylate cyclase type 6-like | **1.82** | -0.33 | Cluster-36535.2 | Cluster-1785.4646 |
|  | vascular endothelial zinc finger 1-like isoform x2 | 0.48 | **-0.47** | Cluster-36583.0 | Cluster-28134.0 |
|  | ceramide kinase-like isoform x2 | **-1.53** | -0.27 | Cluster-36590.0 | Cluster-22027.0 |
|  | beta- -galactosyltransferase 2-like | **-0.98** | 0.66 | Cluster-36601.0 | Cluster-17819.0 |
|  | glycogenin-1-like isoform x1 | -0.19 | **1.50** | Cluster-36691.10348 | Cluster-34545.0 |
|  | serine threonine-protein kinase ulk1-like isoform x2 | **0.38** | **-0.86** | Cluster-36691.10384 | Cluster-36371.0 |
|  | histidine ammonia-lyase | **1.59** | -0.31 | Cluster-36691.10454 | Cluster-30154.0 |
|  | fas-associated death domain protein | **0.60** | 2.06 | Cluster-36691.10539 | Cluster-1785.6919 |
|  | tumor protein p53-inducible nuclear protein 1 | **4.02** | 2.53 | Cluster-36691.11000 | Cluster-1785.3864 |
|  | beta-galactoside-binding lectin-like | -0.81 | **1.14** | Cluster-36691.1106 | Cluster-17437.1 |
|  | cytochrome p450 1b1 | 0.25 | **3.08** | Cluster-36691.1110 | Cluster-7930.0 |
|  | nad-dependent protein deacylase sirtuin- mitochondrial-like | **-0.18** | -1.27 | Cluster-36691.11161 | Cluster-1785.1225 |
|  | hedgehog-interacting protein | **-0.36** | -1.48 | Cluster-36691.11433 | Cluster-19743.0 |
|  | sodium-dependent phosphate transporter 1-b-like | 1.00 | -0.16 | Cluster-36691.11727 | Cluster-35319.0 |
|  | e3 ubiquitin-protein ligase trim21-like | **0.15** | **-1.42** | Cluster-36691.11847 | Cluster-1785.12987 |
|  | epidermal retinol dehydrogenase 2 | **1.24** | -0.06 | Cluster-36691.12028 | Cluster-1785.13284 |
|  | nucleolar protein of 40 kda | 0.21 | **-1.32** | Cluster-36691.12246 | Cluster-20072.0 |
|  | circadian locomoter output cycles protein kaput-like | -0.67 | **-1.69** | Cluster-36691.12293 | Cluster-24148.0 |
|  | serine threonine-protein kinase sgk1 isoform x2 | **3.80** | **0.11** | Cluster-36691.12300 | Cluster-19699.0 |
|  | 11-cis retinol dehydrogenase | **2.16** | 0.03 | Cluster-36691.1246 | Cluster-36475.1 |
|  | delta-1-pyrroline-5-carboxylate mitochondrial | **1.27** | -0.54 | Cluster-36691.12565 | Cluster-1785.12211 |
|  | inhibitor of growth protein 1 | **-0.05** | -1.16 | Cluster-36691.12810 | Cluster-19718.0 |
|  | acyl- synthetase family member mitochondrial-like | -1.76 | **1.22** | Cluster-36691.12912 | Cluster-1785.4149 |
|  | receptor-interacting serine threonine-protein kinase 4 | **-0.93** | 1.34 | Cluster-36691.13123 | Cluster-23935.0 |
|  | sodium-coupled neutral amino acid transporter 2 | **4.00** | 2.30 | Cluster-36691.13153 | Cluster-1785.13238 |
|  | vesicle transport protein sec20-like | 0.31 | -0.93 | Cluster-36691.13237 | Cluster-15760.0 |
|  | d-amino-acid oxidase | **1.37** | -0.65 | Cluster-36691.13276 | Cluster-14427.4 |
|  | patatin-like phospholipase domain-containing protein 2 | 0.34 | 2.48 | Cluster-36691.13455 | Cluster-1785.12483 |
|  | vang-like protein 1 isoform x1 | -0.55 | **0.76** | Cluster-36691.13553 | Cluster-1785.13804 |
|  | beta- -n-acetylglucosaminyltransferase radical fringe | **1.05** | -0.30 | Cluster-36691.13827 | Cluster-1785.7988 |
|  | beta- n-acetylgalactosaminyltransferase 1-like | **0.77** | -1.03 | Cluster-36691.14105 | Cluster-29121.2 |
|  | methyltransferase-like protein 7a | **-0.02** | -1.30 | Cluster-36691.14196 | Cluster-25156.0 |
|  | heparan sulfate glucosamine 3-o-sulfotransferase 3b1-like | **-1.67** | -0.16 | Cluster-36691.14389 | Cluster-32991.0 |
|  | methylthioribose-1-phosphate isomerase | -0.98 | 0.66 | Cluster-36691.14438 | Cluster-19602.0 |
|  | zinc finger protein zic 4-like | **-1.70** | -0.04 | Cluster-36691.14445 | Cluster-17319.0 |
|  | jmjc domain-containing protein 8 | **-2.61** | -0.69 | Cluster-36691.14463 | Cluster-17600.1 |
|  | dis3-like exonuclease 1 | **-0.95** | **0.19** | Cluster-36691.14502 | Cluster-32908.0 |
|  | nuclear receptor subfamily 0 group b member 2 | 0.46 | -2.63 | Cluster-36691.14517 | Cluster-24279.0 |
|  | adp-ribosylation factor-like protein 5b | 1.46 | **3.16** | Cluster-36691.14606 | Cluster-17516.1 |
|  | morc family cw-type zinc finger protein 3 | -0.74 | **0.26** | Cluster-36691.14617 | Cluster-1785.12307 |
|  | phosphatidylinositol 4-phosphate 5-kinase type-1 beta-like | 0.41 | 2.36 | Cluster-36691.14674 | Cluster-1785.2005 |
|  | fibroblast growth factor receptor 4-like | 0.14 | -1.71 | Cluster-36691.14774 | Cluster-33705.0 |
|  | unconventional myosin-xix isoform x1 | **1.11** | -0.73 | Cluster-36691.15182 | Cluster-32548.0 |
|  | px domain-containing protein 1 | **-0.04** | -1.35 | Cluster-36691.1545 | Cluster-19800.0 |
|  | protein yippee-like 3 | -2.75 | **-1.18** | Cluster-36691.15611 | Cluster-1785.12745 |
|  | opsin- partial | 4.28 | **-0.60** | Cluster-36691.15708 | Cluster-31586.0 |
|  | tnf receptor-associated factor 6 | -1.28 | **0.25** | Cluster-36691.15826 | Cluster-34680.0 |
|  | formin-binding protein 1-like isoform x1 | 0.48 | **-0.63** | Cluster-36691.1590 | Cluster-1785.13005 |
|  | metastasis-associated protein mta3 | -1.12 | **0.05** | Cluster-36691.15922 | Cluster-29668.0 |
|  | bis(5 -adenosyl)-triphosphatase enpp4 | **1.29** | -0.30 | Cluster-36691.16083 | Cluster-1785.10337 |
|  | presenilins-associated rhomboid-like mitochondrial | **-1.55** | -0.21 | Cluster-36691.16162 | Cluster-1785.13865 |
|  | serine arginine repetitive matrix protein 1 | **-0.88** | 1.52 | Cluster-36691.16208 | Cluster-15304.2 |
|  | excitatory amino acid transporter 1-like | **-1.67** | **-0.11** | Cluster-36691.16266 | Cluster-1785.10367 |
|  | heparan-alpha-glucosaminide n-acetyltransferase-like | -2.53 | **-0.51** | Cluster-36691.16563 | Cluster-5948.0 |
|  | acyl- synthetase family member mitochondrial-like | **-1.39** | **0.93** | Cluster-36691.17030 | Cluster-1785.4151 |
|  | solute carrier family 13 member 5-like | **0.80** | **-1.01** | Cluster-36691.17386 | Cluster-1785.11515 |
|  | b-cell lymphoma 6 protein isoform x1 | **1.91** | **-0.81** | Cluster-36691.17445 | Cluster-18618.0 |
|  | inositol-3-phosphate synthase 1 | **3.22** | **1.54** | Cluster-36691.17576 | Cluster-1785.2297 |
|  | serine cytosolic | **0.96** | -0.68 | Cluster-36691.17742 | Cluster-29275.0 |
|  | wd repeat and fyve domain-containing protein 1 | 0.20 | **-1.64** | Cluster-36691.17827 | Cluster-1785.8476 |
|  | serine threonine tyrosine-interacting protein | **-0.10** | 1.18 | Cluster-36691.1788 | Cluster-22011.0 |
|  | prkc apoptosis wt1 regulator protein | 0.63 | **-0.43** | Cluster-36691.18057 | Cluster-35278.0 |
|  | canalicular multispecific organic anion transporter 1 | 0.78 | **-0.51** | Cluster-36691.18091 | Cluster-1785.13287 |
|  | paired amphipathic helix protein sin3a-like | -3.28 | **-1.94** | Cluster-36691.18270 | Cluster-1785.9425 |
|  | peroxisome proliferator-activated receptor delta | 0.87 | **-0.54** | Cluster-36691.18457 | Cluster-28971.0 |
|  | vacuolar fusion protein mon1 homolog a | **-0.21** | -1.60 | Cluster-36691.18521 | Cluster-33378.0 |
|  | monocarboxylate transporter 2-like | **-0.16** | -1.35 | Cluster-36691.18523 | Cluster-30258.1 |
|  | sulfotransferase 1c1-like | **-1.95** | 1.13 | Cluster-36691.18534 | Cluster-29327.1 |
|  | activating signal cointegrator 1 complex subunit 1 | 0.46 | -0.95 | Cluster-36691.208 | Cluster-1785.13631 |
|  | phd finger protein 21b isoform x2 | -0.52 | **-1.85** | Cluster-36691.2273 | Cluster-30736.1 |
|  | atp-dependent rna helicase ddx19b | 1.28 | **0.16** | Cluster-36691.2288 | Cluster-10494.0 |
|  | dimethylglycine mitochondrial | **0.80** | -0.98 | Cluster-36691.2366 | Cluster-1785.13445 |
|  | indian hedgehog b | **-0.36** | -1.33 | Cluster-36691.2431 | Cluster-32290.0 |
|  | glutaminase kidney mitochondrial-like | 1.70 | **-1.43** | Cluster-36691.2499 | Cluster-1785.6185 |
|  | procollagen c-endopeptidase enhancer 2 | 1.14 | **-0.40** | Cluster-36691.2519 | Cluster-1785.7076 |
|  | nadph:adrenodoxin mitochondrial | **-1.13** | **-0.09** | Cluster-36691.2761 | Cluster-1785.10787 |
|  | probable c-mannosyltransferase dpy19l1 | **1.23** | **-0.53** | Cluster-36691.2889 | Cluster-32396.0 |
|  | lysine-specific demethylase 4b-like | -0.17 | **-1.46** | Cluster-36691.291 | Cluster-23023.0 |
|  | sarcosine mitochondrial | 1.75 | -0.35 | Cluster-36691.3074 | Cluster-1785.7620 |
|  | wd repeat and socs box-containing protein 2 | **-0.20** | **2.00** | Cluster-36691.3079 | Cluster-1785.13773 |
|  | f-box-like wd repeat-containing protein tbl1x | **1.82** | 0.62 | Cluster-36691.3152 | Cluster-20385.0 |
|  | sulfite mitochondrial | 0.37 | **2.33** | Cluster-36691.3209 | Cluster-1785.3071 |
|  | uridine phosphorylase 2 | 1.87 | **0.17** | Cluster-36691.3381 | Cluster-22485.1 |
|  | trna (uracil-5-)-methyltransferase homolog a | 1.09 | **-0.04** | Cluster-36691.3611 | Cluster-17518.0 |
|  | phosphatidylinositol 4-phosphate 5-kinase type-1 alpha-like | **-1.02** | 0.12 | Cluster-36691.3687 | Cluster-15562.0 |
|  | cingulin isoform x3 | **1.04** | **-0.13** | Cluster-36691.3785 | Cluster-32587.0 |
|  | arsenite methyltransferase-like | 1.08 | **-0.70** | Cluster-36691.394 | Cluster-1842.2 |
|  | ras association domain-containing protein 6 | 0.56 | **-0.64** | Cluster-36691.3945 | Cluster-16186.2 |
|  | glucoside xylosyltransferase 1-like isoform x1 | -0.97 | **-2.55** | Cluster-36691.4075 | Cluster-24087.0 |
|  | transmembrane protein 56-b-like | -1.96 | **-0.02** | Cluster-36691.4150 | Cluster-33335.1 |
|  | transmembrane protein 56-b-like | **-1.15** | 0.35 | Cluster-36691.4151 | Cluster-33335.0 |
|  | rab-like protein 2b | -0.26 | **1.43** | Cluster-36691.418 | Cluster-35601.0 |
|  | l _3 | -4.95 | -0.65 | Cluster-36691.4232 | Cluster-1785.9337 |
|  | e3 ubiquitin-protein ligase xiap-like | **2.19** | 0.90 | Cluster-36691.4275 | Cluster-31084.0 |
|  | eukaryotic translation initiation factor 3 subunit e-b | 0.14 | 1.46 | Cluster-36691.4622 | Cluster-25005.1 |
|  | protein farnesyltransferase geranylgeranyltransferase type-1 subunit alpha | 0.01 | **-1.26** | Cluster-36691.463 | Cluster-1785.8528 |
|  | a disintegrin and metalloproteinase with thrombospondin motifs 6 | -1.13 | **1.19** | Cluster-36691.4640 | Cluster-21266.0 |
|  | gap junction alpha-4 protein | 1.08 | -0.26 | Cluster-36691.4892 | Cluster-32705.0 |
|  | serine threonine-protein kinase rio3 | 2.04 | 0.49 | Cluster-36691.4914 | Cluster-1785.13233 |
|  | run and fyve domain-containing protein 2 isoform x1 | **0.40** | **-0.87** | Cluster-36691.5029 | Cluster-1785.8009 |
|  | krueppel-like factor 11 | **-0.19** | 2.02 | Cluster-36691.5060 | Cluster-1785.1179 |
|  | krueppel-like factor 11 | 0.56 | 2.94 | Cluster-36691.5061 | Cluster-1785.1159 |
|  | gtp-binding protein 1 isoform x1 | -0.26 | **-1.17** | Cluster-36691.5285 | Cluster-29601.0 |
|  | 3-hydroxyacyl- dehydrogenase type-2 | **0.81** | -0.23 | Cluster-36691.5365 | Cluster-1785.11084 |
|  | rna-directed dna polymerase from mobile element jockey-like | -1.01 | **1.18** | Cluster-36691.5403 | Cluster-1785.779 |
|  | solute carrier family 35 member f5 | **-0.80** | 0.62 | Cluster-36691.553 | Cluster-1785.1577 |
|  | acidic leucine-rich nuclear phosphoprotein 32 family member b | -0.97 | **0.31** | Cluster-36691.5596 | Cluster-1785.2703 |
|  | abhydrolase domain-containing protein 4 | 1.46 | **-1.25** | Cluster-36691.5642 | Cluster-23153.0 |
|  | dna helicase ino80 isoform x1 | **-0.50** | 1.06 | Cluster-36691.5916 | Cluster-19790.1 |
|  | protein sprouty homolog 4 | **2.75** | 0.24 | Cluster-36691.5982 | Cluster-21080.0 |
|  | beta-galactosidase | 0.77 | **-0.24** | Cluster-36691.6205 | Cluster-1785.13178 |
|  | long-chain fatty acid transport protein 6 | -0.90 | **0.66** | Cluster-36691.6218 | Cluster-1785.10191 |
|  | mitochondrial 2-oxodicarboxylate carrier | -1.56 | **0.45** | Cluster-36691.6425 | Cluster-31448.0 |
|  | lon peptidase n-terminal domain and ring finger protein 1-like | **1.15** | -0.01 | Cluster-36691.6473 | Cluster-23356.0 |
|  | dual specificity protein phosphatase 13 isoform a-like | -3.88 | **0.29** | Cluster-36691.6494 | Cluster-28610.0 |
|  | induced myeloid leukemia cell differentiation protein mcl-1 homolog | 1.08 | 2.72 | Cluster-36691.6689 | Cluster-1785.7457 |
|  | stromal cell-derived factor 2-like | 1.11 | **-0.84** | Cluster-36691.6751 | Cluster-10772.0 |
|  | phosphoserine phosphatase | -0.50 | 1.52 | Cluster-36691.7152 | Cluster-10303.1 |
|  | neurocalcin-delta b | -1.26 | **1.37** | Cluster-36691.7319 | Cluster-28798.2 |
|  | glycerol-3-phosphate acyltransferase 3-like | **2.26** | -0.11 | Cluster-36691.7479 | Cluster-1785.416 |
|  | ubiquitin-conjugating enzyme e2 variant 3-like | **0.80** | -0.79 | Cluster-36691.7797 | Cluster-1785.10124 |
|  | c-type lysozyme | **0.17** | 3.07 | Cluster-36691.8098 | Cluster-16553.0 |
|  | cyclin-dependent kinase 18 | 0.86 | **-0.70** | Cluster-36691.8307 | Cluster-1785.11714 |
|  | protein tob1-like | **3.08** | 1.47 | Cluster-36691.8317 | Cluster-1785.10229 |
|  | -dihydroxyvitamin d 24- mitochondrial | **2.85** | **0.00** | Cluster-36691.8422 | Cluster-1785.11481 |
|  | 3-hydroxy-3-methylglutaryl-coenzyme a reductase | **1.19** | **-0.92** | Cluster-36691.8589 | Cluster-31593.0 |
|  | lysoplasmalogenase-like | **-0.99** | 0.15 | Cluster-36691.8599 | Cluster-1785.14059 |
|  | polyhomeotic-like protein 2 isoform x1 | 0.16 | -0.79 | Cluster-36691.8722 | Cluster-8991.0 |
|  | dna-directed dna rna polymerase mu | -0.31 | **-2.06** | Cluster-36691.8744 | Cluster-34889.0 |
|  | rna-directed dna polymerase from transposon bs | 0.31 | **2.03** | Cluster-36691.8838 | Cluster-28372.0 |
|  | high mobility group protein b1-like | **3.69** | **1.25** | Cluster-36691.891 | Cluster-21053.0 |
|  | tsc22 domain family protein 3 isoform x1 | **1.07** | -0.39 | Cluster-36691.8926 | Cluster-14345.0 |
|  | 5 -amp-activated protein kinase catalytic subunit alpha-1 | **-0.30** | 0.72 | Cluster-36691.9094 | Cluster-35802.2 |
|  | b-cell lymphoma 6 protein homolog isoform x2 | **1.85** | -1.24 | Cluster-36691.914 | Cluster-8717.1 |
|  | maspardin | **0.44** | -0.80 | Cluster-36691.9367 | Cluster-1785.8085 |
|  | myosin light chain smooth muscle-like isoform x1 | 0.90 | **-0.49** | Cluster-36691.964 | Cluster-1785.13163 |
|  | 4-aminobutyrate mitochondrial | **1.29** | -0.14 | Cluster-36691.9720 | Cluster-21652.0 |
|  | integrator complex subunit 10 isoform x2 | 0.46 | **-1.31** | Cluster-36691.9768 | Cluster-27080.0 |
|  | solute carrier family 12 member 9 | 1.31 | **-0.42** | Cluster-36691.9812 | Cluster-33946.1 |
|  | guanylate kinase isoform x2 | **-0.53** | 0.62 | Cluster-36880.0 | Cluster-28391.0 |
|  | cytosolic 5 -nucleotidase 3a isoform x1 | 2.57 | **0.34** | Cluster-36981.1 | Cluster-32404.0 |
|  | krueppel-like factor 1 | **-1.06** | 0.91 | Cluster-37055.0 | Cluster-12637.0 |
|  | ectonucleoside triphosphate diphosphohydrolase 4 isoform x1 | **-0.05** | 0.91 | Cluster-37097.0 | Cluster-20421.0 |
|  | alkylated dna repair protein alkb homolog 1 | **-1.79** | -0.26 | Cluster-37222.1 | Cluster-33504.1 |
|  | ccaat enhancer-binding protein alpha | **0.03** | 2.06 | Cluster-5453.0 | Cluster-24172.0 |
|  | coiled-coil domain-containing protein 39 | **-0.72** | 1.96 | Cluster-5521.1 | Cluster-1785.11743 |
|  | dihydropyrimidinase-related protein 2 isoform x1 | -1.43 | **1.31** | Cluster-5673.1 | Cluster-26894.0 |
|  | probable atp-dependent rna helicase ddx41 | -0.70 | **2.20** | Cluster-5811.0 | Cluster-26295.0 |
|  | von willebrand factor | **-2.81** | 0.58 | Cluster-5814.0 | Cluster-28546.0 |
|  | cgmp-inhibited 3 -cyclic phosphodiesterase b | 0.79 | **-0.18** | Cluster-6135.0 | Cluster-1785.7540 |
|  | e3 sumo-protein ligase nse2 | **1.37** | -1.01 | Cluster-6242.0 | Cluster-34293.0 |
|  | deoxyribonuclease gamma-like | **4.22** | **-0.09** | Cluster-6529.0 | Cluster-33772.0 |
|  | sphingosine-1-phosphate phosphatase 1-like | **-0.50** | -2.07 | Cluster-6829.0 | Cluster-17923.0 |
|  | beta- -galactosyltransferase 6 | **-1.90** | **-0.01** | Cluster-6866.0 | Cluster-1785.9393 |
|  | myoferlin-like isoform x1 | **-0.83** | 0.89 | Cluster-7026.0 | Cluster-18153.0 |
|  | palmitoyltransferase zdhhc3-like isoform x2 | -0.04 | **-1.65** | Cluster-7127.1 | Cluster-1785.11605 |
|  | popeye domain-containing protein 2 isoform x1 | **0.32** | -1.50 | Cluster-7400.0 | Cluster-8449.1 |
|  | cyclin-dependent kinase 2-associated protein 1 isoform x1 | -2.06 | **-0.45** | Cluster-7535.0 | Cluster-20778.0 |
|  | creatine kinase m-type | -6.19 | 0.64 | Cluster-7774.2 | Cluster-27377.6 |
|  | histone h2a | 4.31 | **2.47** | Cluster-7915.6 | Cluster-1785.5498 |
|  | protein dispatched homolog 1 | **2.02** | 0.91 | Cluster-8090.0 | Cluster-24651.0 |
|  | nuclear receptor subfamily 1 group d member 2 | 2.86 | **0.86** | Cluster-8263.0 | Cluster-36141.1 |
|  | huntingtin-interacting protein 1-related protein | 1.48 | **-0.58** | Cluster-8538.0 | Cluster-31849.0 |
|  | retinol-binding protein 1-like | **2.03** | 0.05 | Cluster-8749.0 | Cluster-1785.12595 |
|  | ras-related protein rab-8b | **0.48** | 1.80 | Cluster-8850.0 | Cluster-29133.0 |
|  | tfiih basal transcription factor complex helicase xpd subunit | 1.01 | **-0.19** | Cluster-8856.2 | Cluster-1785.166 |
|  | transcription factor -like isoform x2 | **-0.48** | -1.53 | Cluster-9754.0 | Cluster-21746.0 |
|  | b-cell cll lymphoma 7 protein family member a isoform x1 | **8.27** | **-0.69** | Cluster-978.0 | Cluster-1785.6797 |
|  | sideroflexin-1 | 0.20 | **-1.33** | Cluster-9790.0 | Cluster-1785.10193 |
|  | phosphatidate phosphatase lpin2-like isoform x2 | 1.60 | -0.40 | Cluster-9852.0 | Cluster-34285.2 |
|  | ets translocation variant 5 isoform x1 | 3.04 | **0.90** | Cluster-9864.5 | Cluster-30082.0 |
|  | csc1-like protein 2 isoform x1 | 1.57 | 0.13 | Cluster-9906.0 | Cluster-35876.0 |

Table S3: Gene Ontology biological process categories that are over-represented by genes differentially transcribed in round goby (RG) and tubenose goby (TNG) liver tissue in response to high and low temperature challenges. Categories are grouped by those represented by genes that are either up or down regulated to indicate turning on or off of biological function. (GO ID: Gene Ontology accession number, DE: number of differentially expressed genes with that GO annotation, Total: total number of genes with that GO annotation, p-value: uncorrected p-value from exact test performed by goseq software, FDR: false discovery rate adjusted p-value).

|  |  | GO ID | Description | DE | Total | p-value | FDR |
| --- | --- | --- | --- | --- | --- | --- | --- |
| **High Temperature** | | |  |  |  |  |  |
|  | *Up regulated* | |  |  |  |  |  |
|  | RG |  |  |  |  |  |  |
|  |  |  |  |  |  |  |  |
|  | TNG | GO:0002252 | immune effector process | 5 | 49 | 1.50E-05 | 2.86E-02 |
|  |  | GO:0002684 | positive regulation of immune system process | 6 | 80 | 1.21E-05 | 2.86E-02 |
|  |  | GO:0002253 | activation of immune response | 5 | 59 | 3.78E-05 | 4.57E-02 |
|  |  | GO:0050778 | positive regulation of immune response | 5 | 62 | 4.80E-05 | 4.57E-02 |
|  |  | GO:0006959 | humoral immune response | 3 | 13 | 7.02E-05 | 4.86E-02 |
|  |  |  |  |  |  |  |  |
|  | *Down regulated* | |  |  |  |  |  |
|  | RG | GO:0022402 | cell cycle process | 32 | 249 | 4.59E-23 | 1.14E-19 |
|  |  | GO:1903047 | mitotic cell cycle process | 27 | 157 | 5.76E-23 | 1.14E-19 |
|  |  | GO:0007049 | cell cycle | 35 | 349 | 1.36E-21 | 1.80E-18 |
|  |  | GO:0000278 | mitotic cell cycle | 27 | 190 | 1.08E-20 | 1.07E-17 |
|  |  | GO:0048285 | organelle fission | 21 | 126 | 1.04E-17 | 8.22E-15 |
|  |  | GO:0000280 | nuclear division | 20 | 111 | 1.27E-17 | 8.39E-15 |
|  |  | GO:0007017 | microtubule-based process | 18 | 170 | 7.79E-12 | 4.41E-09 |
|  |  | GO:0007067 | mitotic nuclear division | 11 | 45 | 1.03E-11 | 5.10E-09 |
|  |  | GO:0006996 | organelle organization | 39 | 957 | 7.90E-11 | 3.47E-08 |
|  |  | GO:1902589 | single-organism organelle organization | 30 | 668 | 2.64E-09 | 7.46E-07 |
|  |  | GO:0000819 | sister chromatid segregation | 7 | 20 | 4.36E-09 | 1.01E-06 |
|  |  | GO:0000070 | mitotic sister chromatid segregation | 7 | 20 | 4.36E-09 | 1.01E-06 |
|  |  | GO:0006261 | DNA-dependent DNA replication | 8 | 32 | 6.31E-09 | 1.39E-06 |
|  |  | GO:0098813 | nuclear chromosome segregation | 7 | 26 | 3.44E-08 | 6.48E-06 |
|  |  | GO:0006259 | DNA metabolic process | 21 | 388 | 3.84E-08 | 6.61E-06 |
|  |  | GO:0051301 | cell division | 10 | 74 | 4.20E-08 | 6.93E-06 |
|  |  | GO:0051276 | chromosome organization | 18 | 295 | 6.45E-08 | 9.83E-06 |
|  |  | GO:0007059 | chromosome segregation | 7 | 29 | 7.82E-08 | 1.15E-05 |
|  |  | GO:0071103 | DNA conformation change | 9 | 61 | 9.25E-08 | 1.31E-05 |
|  |  | GO:0000226 | microtubule cytoskeleton organization | 10 | 85 | 1.62E-07 | 2.14E-05 |
|  |  | GO:0030261 | chromosome condensation | 5 | 12 | 3.01E-07 | 3.73E-05 |
|  |  | GO:0007051 | spindle organization | 7 | 37 | 4.70E-07 | 5.17E-05 |
|  |  | GO:0016043 | cellular component organization | 42 | 1479 | 7.82E-07 | 8.15E-05 |
|  |  | GO:0007346 | regulation of mitotic cell cycle | 8 | 58 | 8.33E-07 | 8.46E-05 |
|  |  | GO:0051726 | regulation of cell cycle | 12 | 154 | 8.57E-07 | 8.48E-05 |
|  |  | GO:1901990 | regulation of mitotic cell cycle phase transition | 7 | 41 | 9.27E-07 | 8.95E-05 |
|  |  | GO:1901987 | regulation of cell cycle phase transition | 7 | 42 | 1.08E-06 | 1.02E-04 |
|  |  | GO:0010564 | regulation of cell cycle process | 9 | 85 | 1.64E-06 | 1.51E-04 |
|  |  | GO:0006260 | DNA replication | 9 | 92 | 3.41E-06 | 2.93E-04 |
|  |  | GO:0071840 | cellular component organization or biogenesis | 42 | 1575 | 4.10E-06 | 3.45E-04 |
|  |  | GO:0007010 | cytoskeleton organization | 15 | 288 | 6.22E-06 | 4.92E-04 |
|  |  | GO:0044772 | mitotic cell cycle phase transition | 7 | 58 | 1.01E-05 | 7.81E-04 |
|  |  | GO:0044770 | cell cycle phase transition | 7 | 60 | 1.27E-05 | 9.31E-04 |
|  |  | GO:0044699 | single-organism process | 98 | 5487 | 1.37E-05 | 9.85E-04 |
|  |  | GO:0010639 | negative regulation of organelle organization | 6 | 44 | 2.18E-05 | 1.54E-03 |
|  |  | GO:0044763 | single-organism cellular process | 83 | 4413 | 2.56E-05 | 1.78E-03 |
|  |  | GO:0033044 | regulation of chromosome organization | 6 | 46 | 2.96E-05 | 2.02E-03 |
|  |  | GO:0010965 | regulation of mitotic sister chromatid separation | 4 | 15 | 3.53E-05 | 2.22E-03 |
|  |  | GO:0033045 | regulation of sister chromatid segregation | 4 | 15 | 3.53E-05 | 2.22E-03 |
|  |  | GO:0033047 | regulation of mitotic sister chromatid segregation | 4 | 15 | 3.53E-05 | 2.22E-03 |
|  |  | GO:1902099 | regulation of metaphase/anaphase transition of cell cycle | 4 | 15 | 3.53E-05 | 2.22E-03 |
|  |  | GO:0071173 | spindle assembly checkpoint | 3 | 6 | 4.44E-05 | 2.63E-03 |
|  |  | GO:0071174 | mitotic spindle checkpoint | 3 | 6 | 4.44E-05 | 2.63E-03 |
|  |  | GO:1904667 | negative regulation of ubiquitin protein ligase activity | 3 | 6 | 4.44E-05 | 2.63E-03 |
|  |  | GO:1904666 | regulation of ubiquitin protein ligase activity | 3 | 6 | 4.44E-05 | 2.63E-03 |
|  |  | GO:0006323 | DNA packaging | 5 | 33 | 6.85E-05 | 3.96E-03 |
|  |  | GO:0051436 | negative regulation of ubiquitin-protein ligase activity involved in mitotic cell cycle | 3 | 7 | 7.54E-05 | 3.96E-03 |
|  |  | GO:0033046 | negative regulation of sister chromatid segregation | 3 | 7 | 7.70E-05 | 3.96E-03 |
|  |  | GO:0033048 | negative regulation of mitotic sister chromatid segregation | 3 | 7 | 7.70E-05 | 3.96E-03 |
|  |  | GO:0045839 | negative regulation of mitotic nuclear division | 3 | 7 | 7.70E-05 | 3.96E-03 |
|  |  | GO:0045841 | negative regulation of mitotic metaphase/anaphase transition | 3 | 7 | 7.70E-05 | 3.96E-03 |
|  |  | GO:0051983 | regulation of chromosome segregation | 4 | 18 | 7.63E-05 | 3.96E-03 |
|  |  | GO:0051985 | negative regulation of chromosome segregation | 3 | 7 | 7.70E-05 | 3.96E-03 |
|  |  | GO:1902100 | negative regulation of metaphase/anaphase transition of cell cycle | 3 | 7 | 7.70E-05 | 3.96E-03 |
|  |  | GO:2000816 | negative regulation of mitotic sister chromatid separation | 3 | 7 | 7.70E-05 | 3.96E-03 |
|  |  | GO:0007088 | regulation of mitotic nuclear division | 4 | 19 | 9.76E-05 | 4.47E-03 |
|  |  | GO:0033043 | regulation of organelle organization | 11 | 209 | 1.04E-04 | 4.67E-03 |
|  |  | GO:0031577 | spindle checkpoint | 3 | 8 | 1.21E-04 | 5.11E-03 |
|  |  | GO:0032435 | negative regulation of proteasomal ubiquitin-dependent protein catabolic process | 3 | 8 | 1.20E-04 | 5.11E-03 |
|  |  | GO:0051782 | negative regulation of cell division | 3 | 8 | 1.23E-04 | 5.11E-03 |
|  |  | GO:0051784 | negative regulation of nuclear division | 3 | 8 | 1.23E-04 | 5.11E-03 |
|  |  | GO:1901799 | negative regulation of proteasomal protein catabolic process | 3 | 8 | 1.20E-04 | 5.11E-03 |
|  |  | GO:0032392 | DNA geometric change | 4 | 21 | 1.46E-04 | 5.95E-03 |
|  |  | GO:0000075 | cell cycle checkpoint | 5 | 40 | 1.69E-04 | 6.77E-03 |
|  |  | GO:1903051 | negative regulation of proteolysis involved in cellular protein catabolic process | 3 | 9 | 1.78E-04 | 6.93E-03 |
|  |  | GO:1903363 | negative regulation of cellular protein catabolic process | 3 | 9 | 1.78E-04 | 6.93E-03 |
|  |  | GO:0030071 | regulation of mitotic metaphase/anaphase transition | 3 | 10 | 2.56E-04 | 9.40E-03 |
|  |  | GO:0032434 | regulation of proteasomal ubiquitin-dependent protein catabolic process | 4 | 25 | 3.01E-04 | 1.08E-02 |
|  |  | GO:0051783 | regulation of nuclear division | 4 | 26 | 3.44E-04 | 1.23E-02 |
|  |  | GO:0042177 | negative regulation of protein catabolic process | 3 | 11 | 3.47E-04 | 1.23E-02 |
|  |  | GO:0051129 | negative regulation of cellular component organization | 6 | 73 | 3.89E-04 | 1.35E-02 |
|  |  | GO:0007091 | metaphase/anaphase transition of mitotic cell cycle | 3 | 12 | 4.48E-04 | 1.50E-02 |
|  |  | GO:0044784 | metaphase/anaphase transition of cell cycle | 3 | 12 | 4.48E-04 | 1.50E-02 |
|  |  | GO:0051302 | regulation of cell division | 5 | 51 | 5.41E-04 | 1.79E-02 |
|  |  | GO:0031145 | anaphase-promoting complex-dependent proteasomal ubiquitin-dependent protein catabolic process | 3 | 13 | 5.71E-04 | 1.86E-02 |
|  |  | GO:0051444 | negative regulation of ubiquitin-protein transferase activity | 3 | 13 | 5.88E-04 | 1.86E-02 |
|  |  | GO:0031397 | negative regulation of protein ubiquitination | 3 | 13 | 5.88E-04 | 1.86E-02 |
|  |  | GO:0051352 | negative regulation of ligase activity | 3 | 13 | 5.88E-04 | 1.86E-02 |
|  |  | GO:0051439 | regulation of ubiquitin-protein ligase activity involved in mitotic cell cycle | 3 | 13 | 5.88E-04 | 1.86E-02 |
|  |  | GO:0031109 | microtubule polymerization or depolymerization | 3 | 14 | 7.30E-04 | 2.30E-02 |
|  |  | GO:0009987 | cellular process | 106 | 6632 | 9.72E-04 | 3.03E-02 |
|  |  | GO:0010948 | negative regulation of cell cycle process | 4 | 34 | 1.02E-03 | 3.14E-02 |
|  |  | GO:2001251 | negative regulation of chromosome organization | 3 | 16 | 1.15E-03 | 3.52E-02 |
|  |  | GO:0070507 | regulation of microtubule cytoskeleton organization | 3 | 17 | 1.31E-03 | 3.96E-02 |
|  |  | GO:0007098 | centrosome cycle | 3 | 17 | 1.35E-03 | 4.02E-02 |
|  |  | GO:0051297 | centrosome organization | 3 | 17 | 1.35E-03 | 4.02E-02 |
|  |  | GO:0061136 | regulation of proteasomal protein catabolic process | 4 | 37 | 1.40E-03 | 4.14E-02 |
|  |  | GO:0031330 | negative regulation of cellular catabolic process | 3 | 18 | 1.63E-03 | 4.79E-02 |
|  |  | GO:0045005 | DNA-dependent DNA replication maintenance of fidelity | 2 | 5 | 1.71E-03 | 4.97E-02 |
|  |  |  |  |  |  |  |  |
|  | TNG | GO:0034660 | ncRNA metabolic process | 12 | 191 | 1.48E-07 | 5.65E-04 |
|  |  | GO:0006418 | tRNA aminoacylation for protein translation | 6 | 38 | 1.09E-06 | 1.08E-03 |
|  |  | GO:0043038 | amino acid activation | 6 | 41 | 1.72E-06 | 1.08E-03 |
|  |  | GO:0043039 | tRNA aminoacylation | 6 | 39 | 1.27E-06 | 1.08E-03 |
|  |  | GO:0006399 | tRNA metabolic process | 7 | 94 | 2.23E-05 | 9.46E-03 |
|  |  | GO:0016072 | rRNA metabolic process | 6 | 78 | 7.31E-05 | 2.78E-02 |
|  |  | GO:0006520 | cellular amino acid metabolic process | 9 | 213 | 1.34E-04 | 4.64E-02 |
|  |  |  |  |  |  |  |  |
| **Low Temperature** | | |  |  |  |  |  |
|  | *Up regulated* | |  |  |  |  |  |
|  | RG | GO:0019752 | carboxylic acid metabolic process | 86 | 398 | 3.48E-08 | 6.88E-05 |
|  |  | GO:0006082 | organic acid metabolic process | 88 | 424 | 1.57E-07 | 2.07E-04 |
|  |  | GO:0043436 | oxoacid metabolic process | 87 | 423 | 2.72E-07 | 2.69E-04 |
|  |  | GO:0044281 | small molecule metabolic process | 148 | 836 | 5.23E-07 | 4.14E-04 |
|  |  | GO:1901575 | organic substance catabolic process | 114 | 610 | 1.15E-06 | 7.59E-04 |
|  |  | GO:0009056 | catabolic process | 125 | 695 | 3.15E-06 | 1.78E-03 |
|  |  | GO:0006787 | porphyrin-containing compound catabolic process | 6 | 7 | 2.09E-05 | 5.17E-03 |
|  |  | GO:0033015 | tetrapyrrole catabolic process | 6 | 7 | 2.09E-05 | 5.17E-03 |
|  |  | GO:0051186 | cofactor metabolic process | 42 | 178 | 1.73E-05 | 5.17E-03 |
|  |  | GO:0051187 | cofactor catabolic process | 8 | 12 | 1.61E-05 | 5.17E-03 |
|  |  | GO:0044257 | cellular protein catabolic process | 54 | 251 | 2.30E-05 | 5.36E-03 |
|  |  | GO:0030163 | protein catabolic process | 56 | 270 | 4.80E-05 | 1.00E-02 |
|  |  | GO:0044248 | cellular catabolic process | 106 | 602 | 5.51E-05 | 1.09E-02 |
|  |  | GO:0006732 | coenzyme metabolic process | 36 | 152 | 6.21E-05 | 1.17E-02 |
|  |  | GO:0015809 | arginine transport | 5 | 6 | 1.49E-04 | 1.34E-02 |
|  |  | GO:0015819 | lysine transport | 5 | 6 | 1.49E-04 | 1.34E-02 |
|  |  | GO:0043090 | amino acid import | 5 | 6 | 1.49E-04 | 1.34E-02 |
|  |  | GO:0043091 | L-arginine import | 5 | 6 | 1.49E-04 | 1.34E-02 |
|  |  | GO:0043092 | L-amino acid import | 5 | 6 | 1.49E-04 | 1.34E-02 |
|  |  | GO:0050896 | response to stimulus | 332 | 2276 | 1.15E-04 | 1.34E-02 |
|  |  | GO:0051603 | proteolysis involved in cellular protein catabolic process | 48 | 230 | 1.39E-04 | 1.34E-02 |
|  |  | GO:0089718 | amino acid import across plasma membrane | 5 | 6 | 1.49E-04 | 1.34E-02 |
|  |  | GO:0090467 | arginine import | 5 | 6 | 1.49E-04 | 1.34E-02 |
|  |  | GO:0098739 | import across plasma membrane | 5 | 6 | 1.49E-04 | 1.34E-02 |
|  |  | GO:1902022 | L-lysine transport | 5 | 6 | 1.49E-04 | 1.34E-02 |
|  |  | GO:1902023 | L-arginine transport | 5 | 6 | 1.49E-04 | 1.34E-02 |
|  |  | GO:1902475 | L-alpha-amino acid transmembrane transport | 5 | 6 | 1.49E-04 | 1.34E-02 |
|  |  | GO:1902765 | L-arginine import into cell | 5 | 6 | 1.49E-04 | 1.34E-02 |
|  |  | GO:1902837 | amino acid import into cell | 5 | 6 | 1.49E-04 | 1.34E-02 |
|  |  | GO:1903400 | L-arginine transmembrane transport | 5 | 6 | 1.49E-04 | 1.34E-02 |
|  |  | GO:1903826 | arginine transmembrane transport | 5 | 6 | 1.49E-04 | 1.34E-02 |
|  |  | GO:2001234 | negative regulation of apoptotic signaling pathway | 8 | 15 | 1.36E-04 | 1.34E-02 |
|  |  | GO:0006165 | nucleoside diphosphate phosphorylation | 12 | 31 | 1.69E-04 | 1.43E-02 |
|  |  | GO:0006757 | ATP generation from ADP | 12 | 31 | 1.69E-04 | 1.43E-02 |
|  |  | GO:0044283 | small molecule biosynthetic process | 35 | 153 | 1.69E-04 | 1.43E-02 |
|  |  | GO:0042221 | response to chemical | 104 | 605 | 1.73E-04 | 1.43E-02 |
|  |  | GO:0009719 | response to endogenous stimulus | 56 | 284 | 1.88E-04 | 1.47E-02 |
|  |  | GO:0019941 | modification-dependent protein catabolic process | 46 | 220 | 1.83E-04 | 1.47E-02 |
|  |  | GO:0051707 | response to other organism | 28 | 114 | 2.13E-04 | 1.62E-02 |
|  |  | GO:0009607 | response to biotic stimulus | 28 | 115 | 2.49E-04 | 1.79E-02 |
|  |  | GO:0043207 | response to external biotic stimulus | 28 | 115 | 2.49E-04 | 1.79E-02 |
|  |  | GO:0043632 | modification-dependent macromolecule catabolic process | 46 | 223 | 2.53E-04 | 1.79E-02 |
|  |  | GO:0072521 | purine-containing compound metabolic process | 42 | 198 | 2.48E-04 | 1.79E-02 |
|  |  | GO:0071103 | DNA conformation change | 18 | 61 | 2.64E-04 | 1.84E-02 |
|  |  | GO:1901605 | alpha-amino acid metabolic process | 33 | 146 | 3.20E-04 | 2.11E-02 |
|  |  | GO:0010033 | response to organic substance | 79 | 444 | 3.53E-04 | 2.22E-02 |
|  |  | GO:0060548 | negative regulation of cell death | 26 | 106 | 3.53E-04 | 2.22E-02 |
|  |  | GO:1901657 | glycosyl compound metabolic process | 37 | 171 | 3.68E-04 | 2.24E-02 |
|  |  | GO:0044710 | single-organism metabolic process | 319 | 2214 | 3.74E-04 | 2.24E-02 |
|  |  | GO:0009628 | response to abiotic stimulus | 35 | 159 | 3.81E-04 | 2.25E-02 |
|  |  | GO:0006090 | pyruvate metabolic process | 12 | 34 | 4.56E-04 | 2.52E-02 |
|  |  | GO:0006520 | cellular amino acid metabolic process | 43 | 210 | 4.48E-04 | 2.52E-02 |
|  |  | GO:0015802 | basic amino acid transport | 5 | 7 | 4.66E-04 | 2.52E-02 |
|  |  | GO:0098657 | import into cell | 5 | 7 | 4.62E-04 | 2.52E-02 |
|  |  | GO:0007623 | circadian rhythm | 9 | 21 | 4.77E-04 | 2.55E-02 |
|  |  | GO:0070887 | cellular response to chemical stimulus | 77 | 437 | 5.68E-04 | 3.00E-02 |
|  |  | GO:0033993 | response to lipid | 24 | 98 | 5.97E-04 | 3.11E-02 |
|  |  | GO:0009119 | ribonucleoside metabolic process | 33 | 151 | 6.21E-04 | 3.14E-02 |
|  |  | GO:0043069 | negative regulation of programmed cell death | 25 | 104 | 6.28E-04 | 3.14E-02 |
|  |  | GO:0071495 | cellular response to endogenous stimulus | 48 | 245 | 6.15E-04 | 3.14E-02 |
|  |  | GO:0006733 | oxidoreduction coenzyme metabolic process | 19 | 71 | 6.78E-04 | 3.31E-02 |
|  |  | GO:0009063 | cellular amino acid catabolic process | 16 | 56 | 7.87E-04 | 3.76E-02 |
|  |  | GO:0009135 | purine nucleoside diphosphate metabolic process | 12 | 36 | 8.16E-04 | 3.76E-02 |
|  |  | GO:0009179 | purine ribonucleoside diphosphate metabolic process | 12 | 36 | 8.16E-04 | 3.76E-02 |
|  |  | GO:0009185 | ribonucleoside diphosphate metabolic process | 12 | 36 | 8.16E-04 | 3.76E-02 |
|  |  | GO:0046031 | ADP metabolic process | 12 | 36 | 8.16E-04 | 3.76E-02 |
|  |  | GO:0044265 | cellular macromolecule catabolic process | 61 | 334 | 8.63E-04 | 3.88E-02 |
|  |  | GO:0044699 | single-organism process | 727 | 5487 | 8.54E-04 | 3.88E-02 |
|  |  | GO:0016051 | carbohydrate biosynthetic process | 14 | 46 | 8.78E-04 | 3.91E-02 |
|  |  | GO:0055086 | nucleobase-containing small molecule metabolic process | 60 | 328 | 9.07E-04 | 3.95E-02 |
|  |  | GO:0015822 | ornithine transport | 4 | 5 | 9.55E-04 | 4.07E-02 |
|  |  | GO:1902253 | regulation of intrinsic apoptotic signaling pathway by p53 class mediator | 4 | 5 | 9.93E-04 | 4.14E-02 |
|  |  | GO:0009396 | folic acid-containing compound biosynthetic process | 4 | 5 | 1.02E-03 | 4.22E-02 |
|  |  | GO:0006261 | DNA-dependent DNA replication | 11 | 32 | 1.04E-03 | 4.23E-02 |
|  |  | GO:0006334 | nucleosome assembly | 5 | 8 | 1.05E-03 | 4.23E-02 |
|  |  | GO:0065004 | protein-DNA complex assembly | 13 | 42 | 1.07E-03 | 4.27E-02 |
|  |  | GO:0002237 | response to molecule of bacterial origin | 5 | 8 | 1.10E-03 | 4.36E-02 |
|  |  | GO:0006952 | defense response | 34 | 163 | 1.28E-03 | 4.89E-02 |
|  |  | GO:0032787 | monocarboxylic acid metabolic process | 31 | 145 | 1.27E-03 | 4.89E-02 |
|  |  | GO:0072524 | pyridine-containing compound metabolic process | 18 | 69 | 1.29E-03 | 4.89E-02 |
|  |  | GO:0010498 | proteasomal protein catabolic process | 28 | 127 | 1.31E-03 | 4.94E-02 |
|  |  |  |  |  |  |  |  |
|  | TNG | GO:0050896 | response to stimulus | 324 | 2185 | 3.34E-10 | 1.27E-06 |
|  |  | GO:0009719 | response to endogenous stimulus | 63 | 275 | 6.64E-09 | 8.74E-06 |
|  |  | GO:0050789 | regulation of biological process | 415 | 3006 | 7.63E-09 | 8.74E-06 |
|  |  | GO:1901700 | response to oxygen-containing compound | 42 | 151 | 9.18E-09 | 8.74E-06 |
|  |  | GO:0009725 | response to hormone | 44 | 167 | 2.48E-08 | 1.82E-05 |
|  |  | GO:0010033 | response to organic substance | 82 | 410 | 2.86E-08 | 1.82E-05 |
|  |  | GO:0065007 | biological regulation | 437 | 3231 | 3.58E-08 | 1.95E-05 |
|  |  | GO:0071495 | cellular response to endogenous stimulus | 55 | 242 | 7.49E-08 | 3.57E-05 |
|  |  | GO:0050794 | regulation of cellular process | 385 | 2810 | 8.52E-08 | 3.61E-05 |
|  |  | GO:0042221 | response to chemical | 103 | 575 | 1.72E-07 | 6.55E-05 |
|  |  | GO:0032870 | cellular response to hormone stimulus | 34 | 129 | 9.38E-07 | 2.98E-04 |
|  |  | GO:0006954 | inflammatory response | 13 | 28 | 2.63E-06 | 6.26E-04 |
|  |  | GO:0010243 | response to organonitrogen compound | 26 | 89 | 2.20E-06 | 6.26E-04 |
|  |  | GO:0051716 | cellular response to stimulus | 261 | 1837 | 2.52E-06 | 6.26E-04 |
|  |  | GO:1901652 | response to peptide | 20 | 59 | 2.61E-06 | 6.26E-04 |
|  |  | GO:1901698 | response to nitrogen compound | 27 | 98 | 4.86E-06 | 1.09E-03 |
|  |  | GO:0070887 | cellular response to chemical stimulus | 75 | 414 | 9.52E-06 | 2.01E-03 |
|  |  | GO:0070613 | regulation of protein processing | 6 | 7 | 1.19E-05 | 2.27E-03 |
|  |  | GO:1903318 | negative regulation of protein maturation | 6 | 7 | 1.19E-05 | 2.27E-03 |
|  |  | GO:0071310 | cellular response to organic substance | 63 | 334 | 1.36E-05 | 2.47E-03 |
|  |  | GO:0043434 | response to peptide hormone | 17 | 51 | 1.86E-05 | 3.09E-03 |
|  |  | GO:1901701 | cellular response to oxygen-containing compound | 26 | 101 | 2.68E-05 | 4.26E-03 |
|  |  | GO:1903317 | regulation of protein maturation | 6 | 8 | 4.33E-05 | 6.60E-03 |
|  |  | GO:0033993 | response to lipid | 27 | 112 | 6.93E-05 | 1.02E-02 |
|  |  | GO:0007623 | circadian rhythm | 9 | 19 | 8.11E-05 | 1.07E-02 |
|  |  | GO:0009987 | cellular process | 783 | 6523 | 8.29E-05 | 1.07E-02 |
|  |  | GO:0010955 | negative regulation of protein processing | 5 | 6 | 9.25E-05 | 1.07E-02 |
|  |  | GO:0023052 | signaling | 216 | 1546 | 8.54E-05 | 1.07E-02 |
|  |  | GO:0044700 | single organism signaling | 215 | 1543 | 1.05E-04 | 1.18E-02 |
|  |  | GO:0048519 | negative regulation of biological process | 103 | 658 | 1.48E-04 | 1.61E-02 |
|  |  | GO:0007154 | cell communication | 218 | 1577 | 1.53E-04 | 1.62E-02 |
|  |  | GO:0009605 | response to external stimulus | 61 | 347 | 1.62E-04 | 1.67E-02 |
|  |  | GO:0071375 | cellular response to peptide hormone stimulus | 14 | 44 | 1.81E-04 | 1.82E-02 |
|  |  | GO:0009611 | response to wounding | 31 | 144 | 2.03E-04 | 1.93E-02 |
|  |  | GO:0007584 | response to nutrient | 6 | 10 | 2.53E-04 | 2.30E-02 |
|  |  | GO:0006952 | defense response | 33 | 160 | 2.82E-04 | 2.44E-02 |
|  |  | GO:0032196 | transposition | 19 | 73 | 2.88E-04 | 2.44E-02 |
|  |  | GO:0071417 | cellular response to organonitrogen compound | 18 | 68 | 3.09E-04 | 2.56E-02 |
|  |  | GO:0009607 | response to biotic stimulus | 24 | 104 | 3.48E-04 | 2.65E-02 |
|  |  | GO:0009628 | response to abiotic stimulus | 33 | 161 | 3.38E-04 | 2.65E-02 |
|  |  | GO:0043207 | response to external biotic stimulus | 24 | 104 | 3.48E-04 | 2.65E-02 |
|  |  | GO:0051707 | response to other organism | 24 | 104 | 3.48E-04 | 2.65E-02 |
|  |  | GO:0019222 | regulation of metabolic process | 219 | 1609 | 3.68E-04 | 2.70E-02 |
|  |  | GO:0044699 | single-organism process | 661 | 5473 | 4.73E-04 | 3.33E-02 |
|  |  | GO:1901653 | cellular response to peptide | 14 | 48 | 4.89E-04 | 3.33E-02 |
|  |  | GO:1901699 | cellular response to nitrogen compound | 19 | 76 | 4.70E-04 | 3.33E-02 |
|  |  | GO:1903506 | regulation of nucleic acid-templated transcription | 123 | 836 | 4.86E-04 | 3.33E-02 |
|  |  | GO:0030509 | BMP signaling pathway | 8 | 19 | 5.46E-04 | 3.59E-02 |
|  |  | GO:0048511 | rhythmic process | 10 | 28 | 5.44E-04 | 3.59E-02 |
|  |  | GO:0007165 | signal transduction | 182 | 1316 | 5.72E-04 | 3.70E-02 |
|  |  | GO:0051252 | regulation of RNA metabolic process | 127 | 872 | 5.92E-04 | 3.72E-02 |
|  |  | GO:0060255 | regulation of macromolecule metabolic process | 180 | 1301 | 6.05E-04 | 3.72E-02 |
|  |  | GO:0014074 | response to purine-containing compound | 5 | 8 | 6.87E-04 | 4.09E-02 |
|  |  | GO:0046683 | response to organophosphorus | 5 | 8 | 6.87E-04 | 4.09E-02 |
|  |  | GO:0010468 | regulation of gene expression | 139 | 972 | 7.05E-04 | 4.14E-02 |
|  |  | GO:1903034 | regulation of response to wounding | 10 | 29 | 7.26E-04 | 4.19E-02 |
|  |  | GO:0031323 | regulation of cellular metabolic process | 185 | 1349 | 7.93E-04 | 4.51E-02 |
|  |  |  |  |  |  |  |  |
|  | *Down regulated* | |  |  |  |  |  |
|  | RG |  |  |  |  |  |  |
|  |  |  |  |  |  |  |  |
|  | TNG |  |  |  |  |  |  |
|  |  |  |  |  |  |  |  |

Table S4: Gene Ontology biological process categories that are over-represented by the most plastic genes (largest 5% of Log_2_ fold change) up regulated by round goby (RG) and tubenose goby (TNG) liver tissue in response to low temperature challenges. (GO ID: Gene Ontology accession number, DE: number of differentially expressed genes with that GO annotation, Total: total number of genes with that GO annotation, p-value: uncorrected p-value from exact test performed by goseq software, FDR: false discovery rate adjusted p-value).

|  | GO ID | Description | DE | Total | p value | FDR |
| --- | --- | --- | --- | --- | --- | --- |
|  |  |  |  |  |  |  |
| RG | GO:0006787 | porphyrin-containing compound catabolic process | 4 | 7 | 4.66E-08 | 9.09E-05 |
|  | GO:0033015 | tetrapyrrole catabolic process | 4 | 7 | 4.66E-08 | 9.09E-05 |
|  | GO:0051187 | cofactor catabolic process | 4 | 12 | 6.22E-07 | 8.10E-04 |
|  | GO:0009628 | response to abiotic stimulus | 8 | 159 | 5.76E-06 | 4.08E-03 |
|  | GO:0042168 | heme metabolic process | 4 | 20 | 6.28E-06 | 4.08E-03 |
|  | GO:0046173 | polyol biosynthetic process | 3 | 7 | 9.05E-06 | 4.87E-03 |
|  | GO:0016051 | carbohydrate biosynthetic process | 5 | 46 | 9.98E-06 | 4.87E-03 |
|  | GO:0009755 | hormone-mediated signaling pathway | 5 | 50 | 1.40E-05 | 6.08E-03 |
|  | GO:0034637 | cellular carbohydrate biosynthetic process | 4 | 25 | 1.71E-05 | 6.11E-03 |
|  | GO:0006778 | porphyrin-containing compound metabolic process | 4 | 26 | 1.88E-05 | 6.11E-03 |
|  | GO:0033013 | tetrapyrrole metabolic process | 4 | 29 | 2.89E-05 | 8.05E-03 |
|  | GO:0006020 | inositol metabolic process | 3 | 11 | 3.80E-05 | 9.90E-03 |
|  | GO:0042440 | pigment metabolic process | 4 | 34 | 5.66E-05 | 1.38E-02 |
|  | GO:0071383 | cellular response to steroid hormone stimulus | 5 | 71 | 7.64E-05 | 1.75E-02 |
|  | GO:0036293 | response to decreased oxygen levels | 4 | 42 | 1.25E-04 | 2.68E-02 |
|  | GO:0070482 | response to oxygen levels | 4 | 43 | 1.38E-04 | 2.68E-02 |
|  | GO:0071396 | cellular response to lipid | 5 | 81 | 1.44E-04 | 2.68E-02 |
|  | GO:0048545 | response to steroid hormone | 5 | 81 | 1.44E-04 | 2.68E-02 |
|  | GO:0071407 | cellular response to organic cyclic compound | 5 | 83 | 1.61E-04 | 2.86E-02 |
|  | GO:0046483 | heterocycle metabolic process | 26 | 2115 | 2.09E-04 | 3.40E-02 |
|  | GO:0046700 | heterocycle catabolic process | 6 | 149 | 3.08E-04 | 4.42E-02 |
|  | GO:0007623 | circadian rhythm | 3 | 21 | 3.09E-04 | 4.42E-02 |
|  | GO:0044262 | cellular carbohydrate metabolic process | 5 | 96 | 3.34E-04 | 4.42E-02 |
|  | GO:1901360 | organic cyclic compound metabolic process | 26 | 2174 | 3.34E-04 | 4.42E-02 |
|  | GO:0046165 | alcohol biosynthetic process | 3 | 22 | 3.45E-04 | 4.42E-02 |
|  | GO:0034641 | cellular nitrogen compound metabolic process | 26 | 2180 | 3.48E-04 | 4.42E-02 |
|  | GO:0033993 | response to lipid | 5 | 98 | 3.51E-04 | 4.42E-02 |
|  | GO:0006006 | glucose metabolic process | 3 | 23 | 4.01E-04 | 4.89E-02 |
|  |  |  |  |  |  |  |
| TNG | GO:0046173 | polyol biosynthetic process | 3 | 6 | 3.33E-06 | 1.26E-02 |
|  | GO:0030148 | sphingolipid biosynthetic process | 3 | 10 | 1.98E-05 | 2.14E-02 |
|  | GO:0097164 | ammonium ion metabolic process | 4 | 30 | 2.27E-05 | 2.14E-02 |
|  | GO:0006672 | ceramide metabolic process | 3 | 14 | 5.85E-05 | 3.69E-02 |
|  | GO:0046165 | alcohol biosynthetic process | 3 | 14 | 5.88E-05 | 3.69E-02 |
|  |  |  |  |  |  |  |
